# Neoadjuvant immunochemotherapy for locally advanced resectable oral squamous cell carcinoma: a prospective single-arm trial (Illuminate Trial)

**DOI:** 10.1097/JS9.0000000000000489

**Published:** 2023-06-05

**Authors:** Yingying Huang, Jingjing Sun, Jun Li, Dongwang Zhu, Minjun Dong, Shengjin Dou, Yong Tang, Wentao Shi, Qi Sun, Tongchao Zhao, Zhihang Zhou, Xinyu Zhou, Ying Liu, Jiang Li, Guopei Zhu, Ding Zhang, Yanan Chen, Qi Zhu, Wutong Ju, Laiping Zhong

**Affiliations:** a Department of Oral and Maxillofacial-Head and Neck Oncology; b Department of Oral Pathology, Ninth People’s Hospital, College of Stomatology; c Department of Radiology; d Huangpu Branch; e Department of Biostatistics in Clinical Research Unit, Ninth People’s Hospital, Shanghai Jiao Tong University School of Medicine; f The Medical Department, 3D Medicines Inc; g National Center for Stomatology; h National Clinical Research Center for Oral Diseases; i Shanghai Key Laboratory of Stomatology, Shanghai, People’s Republic China

**Keywords:** major pathological response, neoadjuvant immunochemotherapy, oral squamous cell carcinoma, PD-1 inhibitor

## Abstract

**Background:**

Locally advanced oral squamous cell carcinoma (LAOSCC) is associated with a high rate of recurrence and poor survival. Given the recent successes of neoadjuvant immunochemotherapy (NAICT) in solid tumors, it is promising to use this treatment modality to achieve a better pathological response and improve the survival of LAOSCC, and clinical evidence is needed to assess its safety and efficacy.

**Patients and Methods:**

A prospective trial of NAICT with toripalimab (PD-1 inhibitor) and albumin paclitaxel/cisplatin (TTP) was conducted in patients with clinical stage III and IVA OSCC. Intravenous albumin paclitaxel (260 mg/m^2^), cisplatin (75 mg/m^2^), and toripalimab (240 mg) were given in sequence on day 1 of each 21 day cycle for two cycles, followed by radical surgery and risk-adapted adjuvant (chemo)radiotherapy. The primary endpoints were safety and major pathological response (MPR). Targeted next generation sequencing and multiplex immunofluorescence were performed to assess clinical molecular characteristics and the tumor immune microenvironment in the pre-NAICT and post-NAICT tumor samples.

**Results:**

Twenty patients were enrolled. NAICT was well-tolerated with a low incidence of grades 3–4 adverse events in three patients. The completion rates of NAICT and subsequent R0 resection were 100%. The MPR rate was 60%, including a 30% pathological complete response. MPR was achieved in all four patients with a combined positive score of PD-L1>10. The density of tertiary lymphatic structure in post-NAICT tumor samples predicted the pathological response to NAICT. During the median 23-month follow-up, the disease-free survival was 90%, and the overall survival was 95%.

**Conclusions:**

NAICT with the TTP protocol in LAOSCC is feasible and well tolerated, with a promising MPR and no obstruction on subsequent surgery. This trial is supportive of further randomized trials using NAICT in LAOSCC.

## Introduction

Highlights

- Neoadjuvant immunochemotherapy (NAICT) in patients with locally advanced resectable oral cancer was feasible and well tolerated, with no new safety signals.
- The completion rates of NAICT and R0 resection were 100%.
- The major pathological response rate was 60% and 2-year overall survival was 95%.
- The density of tertiary lymphatic structure in post-NAICT tumor samples predicted the pathological response to NAICT.

Oral squamous cell carcinoma (OSCC) is one of the most common cancers of the head and neck region. More than 60% of OSCC patients are at late clinical stages at the first clinic visit, and their 5-year survival rates are less than 50%^1^. Despite the current availability of multiple traditional therapeutic strategies and immunotherapy, ~50% of patients suffer local recurrence or distant metastasis^2,3^. The lack of significant improvement in survival and quality of life highlights the urgent need for novel treatment strategies for OSCC.

Neoadjuvant therapy with clinically validated antitumor activity has driven many clinical trials in solid tumors^4–7^. The theoretical purposes of neoadjuvant therapy include reducing the burden of locoregional disease, thus facilitating surgical resection and the elimination of minimal residual tumor, which reduce the risk of distant metastases and allow symptomatic improvements that can result in increased performance^4^. Major pathological response (MPR) to neoadjuvant therapy, which is defined as less than or equal to 10% residual viable tumor cells in the primary tumor bed after neoadjuvant therapy, has been validated to predict longer survival^8^, so it has been recommended as an alternative endpoint for neoadjuvant clinical trials in many solid tumors, including OSCC^8–11^.

Immune checkpoint inhibitors have been demonstrated to improve survival in the first-line and second-line settings of recurrent/metastatic head and neck squamous cell carcinoma (HNSCC)^12–14^. These successes have led to immune checkpoint inhibitors being tested in the neoadjuvant setting for the treatment of locally advanced HNSCC, including OSCC. However, neoadjuvant antiprogrammed cell death 1(PD-1) monotherapy has been reported to elicit a relatively low MPR rate (6% for pembrolizumab and 8% for nivolumab) in OSCC^15–17^. Preclinical data suggest that adding chemotherapy to PD-1 inhibitor therapy can increase antigen release due to tumor cell death, which stimulates T-cell-mediated immunity; furthermore, several clinical trials have shown that neoadjuvant immunochemotherapy (NAICT) results in significantly longer survival and a higher percentage of patients with a pathological complete response (pCR) in solid tumor^18–21^. However, there is insufficient clinical evidence in support of an improved pathological response rate to NAICT in OSCC.

In this study, we conducted a prospective, single-arm trial to assess the safety and pathological efficacy of NAICT with toripalimab (PD-1 inhibitor) and albumin paclitaxel/cisplatin (TTP) in patients with locally advanced resectable OSCC (LAROSCC); the completion of NAICT, R0 resection of the primary tumor after NAICT and predictive biomarkers of pathological response were also investigated.

## Methods

### Study design and participants

This trial was a prospective, single-arm, open-label trial. This work has been reported in line with the strengthening the reporting of cohort, cross-sectional and case-control studies in surgery (STROCSS) criteria^22^, Supplemental Digital Content 1, http://links.lww.com/JS9/A569. The key inclusion criteria were patients aged 18 to 75 years with a histopathological diagnosis of OSCC of clinical stages III or IVA (AJCC, 8^th^ edition), an ECOG PS of 0 to 1, and adequate organ and bone marrow function. The key exclusion criteria included prior systemic cancer therapy, known immunosuppression, active autoimmune disease, or any condition that could interfere with trial participation. The full protocol is provided in Supplemental Digital Content (SDC), Supplemental Digital Content 2, http://links.lww.com/JS9/A570. Each patient signed informed consent before participating in this trial.

### Treatment

**Figure 1:**
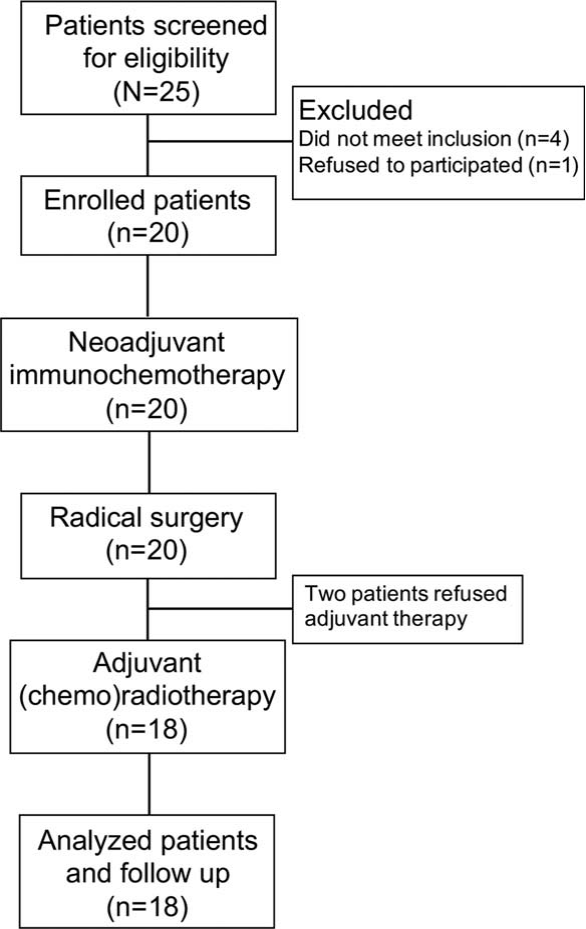
Patient screening and disposition flow diagram.

As shown in the trial flowchart (Fig. 1), the enrolled patients received two cycles of NAICT (21 day each cycle), including intravenous albumin paclitaxel (260 mg/m^2^), cisplatin (75 mg/m^2^), and toripalimab (240 mg) in sequence on day 1 of each cycle. Dose discontinuations, interruptions, and modifications of NAICT were permitted when hyper progressive disease (PD) or unacceptable toxic reactions occurred. Radical surgery was performed within 2 weeks after two cycles of NAICT. The surgical scope was determined by the size of lesions at baseline according to previous neoadjuvant trials^23,24^. The pre-NAICT tumor bed was recorded by photographing and radiographic examination. The initial primary lesion margins (both the longest and shortest axes) were marked by at least four points with tattoo ink, which were 0.5 cm away from the palpable lesion margins. Even if the post-NAICT tumor shrank, the resection range was determined by the pre-NAICT tumor bed, so surgical margins were 0.5–1.0 cm away from the markers; initial intraoral photographs and imaging findings were also used as references (Supplementary Figure 1, Supplemental Digital Content 2, http://links.lww.com/JS9/A570). For example, in patient No. 10, we connected the original marking points as references for the determination of surgical safety margins (0.5–1.0 cm away from the marking points) and surgical tumor bed (Supplementary Figure 2, Supplemental Digital Content 2, http://links.lww.com/JS9/A570). Risk-adapted adjuvant (chemo)radiotherapy was planned within 4–6 weeks after surgery.

### Assessments

Pathological response to NAICT of primary tumors were assessed on hematoxylin and eosin (HE) staining, and it was showed with the proportion of patients with a MPR, including pCR, which was defined as the absence of any tumor cells^8^. Post-NAICT pathological response pattern was described. Taking breast cancer as a reference, pathological response patterns to neoadjuvant therapy were classified as unifocal or multifocal regression^25^. The detailed was shown in SDC, Additional Study Methods. Radiographical response to NAICT was performed according to the RECIST v1.1^26^. Neoadjuvant and adjuvant therapy-related adverse events (AEs) were recorded throughout the study and graded per the NCI-CTCAE v5.0^27^. Surgery-related complications were graded per the Clavien–Dindo Classification^28^.

### Study endpoints

The primary endpoints were safety and MPR. The secondary endpoints were the 2-year overall survival (OS; time from enrollment to death from any cause) and disease-free survival [(DFS); time from enrollment to pathologically confirmed disease recurrence/metastasis or death from any cause].

### Biomarker analyses

Biomarker analyses were performed to evaluate pathological response according to tumor PD-L1 expression, tumor mutational burden (TMB), and tumor-infiltrating lymphocytes (TILs) in the tumor immune microenvironment (TIME). PD-L1 expression in tumor biopsy samples was detected and evaluated by certified pathologists using IHC staining with the 22C3 pharmDx assay. The combined positive score (CPS) was defined as the total number of PD-L1-stained cells (including tumor cells, tumor-associated lymphocytes, and macrophages) divided by the total viable tumor cells multiplied by 100. TMB and genetic analysis were evaluated by targeted next generation sequencing. Multiplex immunofluorescence (MIF) was performed to evaluate TILs. Further details on the biomarker assays and analysis are outlined in SDC, Additional Study Methods.

### Statistical analysis

A sample size of 20 evaluable patients was required to achieve 90% power to detect an increase in the MPR rate from 7% (anti-PD-1 monotherapy, on the basis of data from pembrolizumab and nivolumab monotherapy) to 30%^15,17^, with a one-sided exact test and a significance level of 0.05.

For descriptive analyses, categorical data are expressed as numbers and percentages. Time to-event analyses were summarized using Kaplan–Meier methodology. Comparisons of biomarker subgroups were performed using a *χ* ^2^, Fisher exact, or unpaired Mann–Whitney/Wilcoxon rank-sum tests.

## Results

### Patients and treatment delivery

Between November 2020 and April 2021, 25 patients were screened, among whom 20 were enrolled in this trial. There were 16 patients at clinical stage III and four patients at stage IVA (Table 1). The median age of patients was 54 years. The tongue was the most common primary site (detailed in SDC, Supplementary Table 1, Supplemental Digital Content 2, http://links.lww.com/JS9/A570).

**Table 1:** Baseline patient demographic and clinical characteristics.

Characteristics	*N* (%)
Sex	
Male	16 (80)
Female	4 (20)
Age, years	
Median (IQR)	54 (45–63.8)
Range	19–75
ECGO PS	
0	9 (45)
1	11 (55)
Smoking status	
Current or former	14 (70)
Never	6 (30)
Alcohol use	
Current or former	14 (70)
Never	6 (30)
BMI	
Median (IQR)	23.03 (21.6–25.2)
Range	16.90–30.67
Primary site	
Tongue	8 (40)
Mouth floor	6 (30)
Gingiva	3 (15)
Retromolar trigone	2 (10)
Buccal	1 (5)
TNM stage (AJCC 8th edition)	
T3	16 (80)
T4a	4 (20)
N0	13 (65)
N1	7 (35)
M0	20 (100)
Clinical stage	
III	16 (80)
IVA	4 (20)
Combined positive score	
CPS<1	12 (60)
1≤CPS<10	4 (20)
CPS≥10	4 (20)

All data are represented as *N* (%), unless otherwise specified.

CPS, combined positive score; ECGO PS, eastern cooperative oncology group performance status; IQR, interquartile range; *N* (%), number (percentage).

All patients received two cycles of NAICT and subsequent radical surgery. The completion rate for NAICT and surgery was 100%. Three patients postponed the second cycle for no more than 3 days because of COVID-19 epidemic control. During NAICT, no hyper PD or unacceptable toxic reactions occurred. Eighteen patients received radical surgery on time, while one patient delayed surgery for one day because of insurance, and one patient delayed surgery for 6 days because of personal preference. The median time from initiating NAICT to surgery was 47 (range: 42–61) days. Eighteen patients received postoperative adjuvant (chemo)radiotherapy, while two patients refused adjuvant treatment because of personal preference.

### Safety

The occurrence rates of different degrees of treatment-related AEs are listed in Table 2, and all AEs were relieved after expectant treatment. No AEs led to discontinuation of the whole treatment, dose reduction, delayed surgery, or death.

**Table 2:** Treatment-Related adverse events.

AEs, *N* (%)	All	Grade 1–2	Grade 3	Grade 4
NAICT-related AEs				
Baldness	20 (100)	20 (100)	0	0
Nausea/vomiting	14 (70)	13 (65)	1 (5)	0
Fatigue	12 (60)	12 (60)	0	0
Neutropenia	9 (45)	7 (35)	1 (5)	1 (5)
Leukopenia	8 (40)	6 (30)	1 (5)	1 (5)
Skin (rash, dryness, dermatitis)	8 (40)	7 (35)	1 (5)	0
Diarrhea	8 (40)	8 (40)	0	0
Hyperbilirubinemia	7 (35)	7 (35)	0	0
Uric acid elevation	7 (35)	7 (35)	0	0
Hypokalemia	6 (30)	5 (25)	1 (5)	0
Pain (joint and muscle)	6 (30)	6 (30)	0	0
Proteinuria	6 (30)	6 (30)	0	0
AST elevation	3 (15)	3 (15)	0	0
ALT elevation	3 (15)	3 (15)	0	0
Hyperthyroidism	1 (5)	1 (5)	0	0
Surgery-related AEs				
Subcutaneous exudate	2 (10)	2 (10)	0	0
Flap necrosis	1 (5)	0	1 (5)	0
Flap distal partial necrosis	1 (5)	1 (5)	0	0
Adjuvant treatment-related AEs				
Radiation-induced oral mucositis	18 (90)	18 (90)	0	0
Radiation-induced dermatitis	18 (90)	18 (90)	0	0
Fever	7 (35)	7 (35)	0	0
Hemoglobin decreased	7 (35)	7 (35)	0	0
Leukopenia	6 (30)	6 (30)	0	0
Neutropenia	3 (15)	3 (15)	0	0

Adverse events were graded according to National Cancer Institute Common Terminology Criteria (CTCAE) version 5.0.All data are represented as *N* (%), unless otherwise specified.

AEs, adverse events; ALT, alanine aminotransferase; AST, aspartate aminotransferase; NAICT, neoadjuvant immunochemotherapy; TRAEs, treatment-related adverse events.

During NAICT, grade 3/4 AEs occurred in three patients (15%) including grade 4 neutropenia (5%), grade 3 leukopenia (10%), and grade 3 neutropenia, hypokalemia, vomiting, and rash (5%). Other most common grade 1/2 AEs were baldness (100%), nausea/vomiting (70%), and fatigue (60%) (Table 2). Surgical-related AEs included grade 3a flap necrosis (5%), grade 1 subcutaneous exudation (10%), and grade 1 distal skin flap necrosis (5%). No adjuvant (chemo)radiotherapy-related AEs above grade 3 occurred, and grade 1/2 AEs included oral mucositis and dermatitis (Table 2). All surgery-related and adjuvant (chemo)radiotherapy-related AEs were unrelated to NAICT. Detailed treatment-related AEs are presented in SDC, Supplementary Table 2, Supplemental Digital Content 2, http://links.lww.com/JS9/A570.

### Efficacy of NAICT

**Figure 2:**
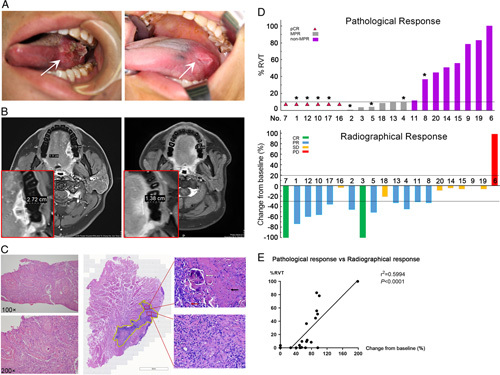
Pathological and radiographical response to NAICT. (A) Tumor regression in a 31-year-old man with clinical stage III tongue cancer (T3N0M0, Patient No. 10). Pre-NAICT image showed a crateriform lesion on left lingual margin (left, white arrow); Post-NAICT image showed tumor shrank significantly (right, white arrow). (B) MR imaging showed the longest diameter of lesion was 2.72 cm in pre-NAICT (left) and 1.38 cm in post-NAICT (right). (C) Pathological findings of the biopsy specimen with hematoxylin–eosin staining, magnifications, 100 and 200 confirmed squamous cell carcinoma (left). Histopathologic images of the post-NAICT resection specimen showed pathological tumor bed (area marked by yellow line) with necrosis (black arrow), residual cornified pearl (white arrow), multinucleated giant cell infiltration (red arrow) and proliferative fibrosis (lower right), with no residual viable tumor (pathological complete response). (D) Waterfall plots showed pathological response and radiographical response to NAICT of individual patients who completed NAICT and surgery (*n*=20). Asterisks represents patients with CPS>1. (E) The pathological and radiographical response to NAICT was consistent in linear regression analysis (*P*<0.001).

All 20 patients were evaluable for pathological and radiographical response to NAICT (patient No. 10 was taken as an example in Fig. 2A–C) (detailed in SDC, Supplementary Table 3, Supplemental Digital Content 2, http://links.lww.com/JS9/A570). According to pathological evaluation, the MPR rate was 60% (12/20) (95% CI: 36.41–80.02%), including 30% (6/20) (95% CI:12.84–54.33%) with pCR. According to RECIST v1.1, two patients (10%) achieved CR and ten (50%) PR; the objective response rate was 60% (12/20) (95% CI: 36.41–80.02%). Only one (5%) had PD. Waterfall plots of pathological and radiographical responses are shown in Figure 2D. Pathological and radiographical responses were significantly consistent in linear regression analysis (*P<*0.001, Fig. 2E).

Regarding regional lymph nodes (LNs), pathological regression was found in four of six patients (11 metastatic nodes in total) and was characterized by the presence of necrosis, multinucleated giant cells, and calcification. Only two nodes achieved MPR (including one at pCR level), and these occurred in one patient (No. 16 patient) who achieved pCR in the primary tumor (SDC, Supplementary Table 4, Supplemental Digital Content 2, http://links.lww.com/JS9/A570).

Clinical downstaging after NAICT occurred in 50% (10/20) (95% CI: 27.85–72.51%) of patients. Clinical upstaging after NAICT occurred in one patient. The pathological stage was not exactly the same as the clinical stage after NAICT. The clinical to pathological downstaging rate of NAICT was 45% (9/20) (95% CI: 23.83–67.95%) (SDC, Supplementary Table 5, Supplemental Digital Content 2, http://links.lww.com/JS9/A570).

### Pathological characteristics

HE staining of post-NAICT samples revealed that the pathological tumor bed was characterized by immune activation-dense TILs, massive tumor cell death with cholesterol clefts, and tissue repair with proliferative fibrosis (SDC, Supplementary Figure 4, Supplemental Digital Content 2, http://links.lww.com/JS9/A570). After systematically reviewing all resected specimens, we found that the most common pattern of tumor response to NAICT was the multifocal regression pattern (SDC, Supplementary Figure 5A, Supplemental Digital Content 2, http://links.lww.com/JS9/A570), which was seen in 92.9% (13/14) of patients, except the patients who achieved pCR. Unifocal regression was found in only one patient with non-MPR (SDC, Supplementary Figure 5B, Supplemental Digital Content 2, http://links.lww.com/JS9/A570). In the MPR group, pathological tumor bed regression led to large areas without residual tumor cells, and microscopic residual foci could be found deep inside the lesion and even close to the margin.

### Biomarker analysis

**Figure 3:**
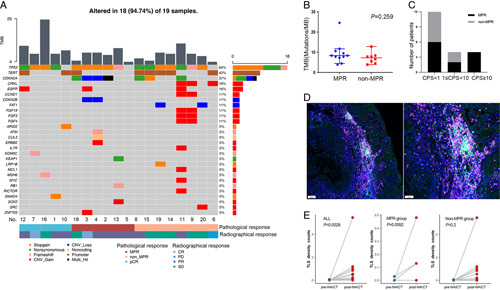
Biomarker analysis of pathological response to NAICT. (A) Oncoplot showing mutations as assessed with a customized NGS panel targeting 733 cancer-related genes of baseline primary tumor samples (*n*=19; 5 pCR, 6 MPR, 8 non-MPR); A column represents a patient. Top bar chart represents tumor mutational burden (TMB). Percentages listed right represent the proportion of samples harboring a mutation in the gene listed left. Patients No. are listed at bottom. Bottom bars show pathological response and radiographical response. (B) The difference of TMB between the MPR and non-MPR groups is not statistically significant in unpaired t test (*P*=0.26). (C) The relationship between CPS and pathological response to NAICT is analyzed, based on the different CPS cutoffs (1, 10), all four patients with CPS>10 achieved MPR, and patients in the CPS>1 group had a higher MPR rate (6/8, 75%) than those in the CPS<1 group (6/12, 50%), but the difference between the two groups is not statistically significant in the Fisher’s exact test (*P*=0.423). (D) The post-NAICT sample with MIF for TILs staining showing TLS formation, which is composed of CD8+ T cell (pink fluorescent) and CD20+ B cell (green fluorescent) (the sample from patient No.18; left, 100×; right, 200×; DAPI, blue fluorescent). (E) More TLS was found in the post-NAICT samples compared with the pre-NAICT samples (*P*=0.0028), the difference is statistically significant in the MPR group (*P*=0.0092), not in the non-MPR group in Wilcoxon signed-rank test (*P*=0.20).

NGS was performed in 19 patients. The most frequently mutated gene was *TP53* (16/19, 84%). Copy number gain of *CRKL*, *CCND1*, and *FGF19/3/4* and copy number loss of *FAT1* only occurred in the non-MPR group. Copy number loss of *CDKN2B* only occurred in the MPR group (Fig. 3A). Patients with higher TMB tended to achieve MPR, but the difference in TMB between the MPR and non-MPR groups was not significant (*P*=0.259, Fig. 3B).

The relationship between CPS and the pathological response to NAICT was analyzed. Based on the different CPS cutoffs (1, 10), all four patients with CPS greater than 10 achieved MPR, and patients in the CPS greater than or equal to 1 group had a higher MPR rate (6/8, 75%) than those in the CPS less than 1 group (6/12, 50%), but the difference between the two groups was not statistically significant (*P*=0.423, Fig. 3C).

According to MIF for TILs staining, the density and spatial distribution of TILs in the TIME were analyzed. More tertiary lymphatic structure (TLS) was found in post-NAICT samples than in pre-NAICT samples (*P*=0.0028), and the difference was significant in the MPR group (*P*=0.009) but not in the non-MPR group (*P*=0.2) (Fig. 3D, E). In pre-NAICT samples, compared with the non-MPR group, tissues from the MPR group were infiltrated with more CD8+ (*P*=0.054), CD68+CD163− (*P*=0.09), cells in the tumor region and CD20+ (*P*=0.059) and CD3+ (*P*=0.098) cells in the stroma region. (SDC, Supplementary Figure 6, Supplemental Digital Content 2, http://links.lww.com/JS9/A570).

### Outcomes

As of February 2023, all patients had been followed up for between 23 and 27 months, with a mean follow-up time of 24 months. One patient died of acute hemorrhage of the upper digestive tract 4 months after treatment; the death was assessed as unrelated to OSCC. Lymph node metastasis occurred in one patient 8 months after treatment. One patient experienced esophageal squamous cell cancer 1-year after treatment and was diagnosed with a second primary cancer. To date, the estimated 2-year OS rate is 95%, and the DFS rate is 90%. The OS curve and DFS curve are shown in SDC, Supplementary Figure 7, Supplemental Digital Content 2, http://links.lww.com/JS9/A570.

## Discussion

The results in our trial demonstrate that NAICT with the TTP protocol is well tolerated with no unexpected treatment-related toxicities in LAROSCC. The completion rate of NAICT and subsequent surgery was 100%, and the pathological response to NAICT was satisfactory with a 60% MPR.

The safety of neoadjuvant therapy and whether it will delay the subsequent surgery have always been a concern for clinicians. The incidence of grade 3/4 AEs in neoadjuvant chemotherapy with the TPF regimen ranges from 9 to 38%^22,28^, and these AEs sometimes delay surgical treatment. In our trial, only three patients (15%) suffered grade 3/4 neoadjuvant therapy-related AEs, and no AEs led to discontinuation of the whole study, dose reduction, surgery delay, or death. In contrast, the all-cause grade 3 or higher toxicity rate was higher in the KEYNOTE-048 trial with immunochemotherapy in R/M HNSCC^13^. We propose that the patient’s physical condition is more tolerant to treatment in the neoadjuvant setting than in the adjuvant setting. Although surgery-related AEs were observed in this study, these were considered unrelated to neoadjuvant therapy; however, trials with larger sample sizes are necessary to definitively indicate the safety of NAICT and its effect on surgery.

As a better pathological response to neoadjuvant therapy predicts a better prognosis, the rate of MPR has been proposed to be a crucial criterion for clinical trials of neoadjuvant therapy in OSCC or HNSCC^11^. The neoadjuvant TTP protocol has shown promising MPR rates (60%), compared with neoadjuvant chemotherapy with PF (33%) or TPF (27.7%) in OSCC^23,29^, and compared with pembrolizumab monotherapy (6%) and nivolumab monotherapy (8%) in HNSCC^15–17^. In our previous study of neoadjuvant therapy using a chemo-free combination of camrelizumab and apatinib in OSCC, the MPR rate was 40%^24^. In contrast, the pathological response to NAICT in LAROSCC per our trial was satisfactory, although cross-study comparisons should be made with caution.

The inconsistent pathological response to NAICT between the primary sites and metastatic LNs is observed in our trial. The MPR rate of the primary sites is as high as 60%; however, that of the LNs is much lower (one in six patients; two in 11 nodes). In a pooled analysis of lung cancer with NAICT, the pCR rate is 40.1% in the primary sites and 62.5% in the metastatic LNs^30^. A neoadjuvant anti-PD-1 therapy in HNSCC also reported a better pathological response in the LNs than in the primary sites^31^. In comparison, the small sample size prevents us from obtaining a definitive conclusion. Besides, the assessment procedures of immune-related pathological response in LNs have not been well defined, and the evaluation of the exact percentage of viable tumor cells in LNs is scarcely reported. Therefore, more attention should be paid on the pathological response in LNs for neoadjuvant treatment studies.

Different degrees of primary tumor regression have been reported in LAROSCC treated with neoadjuvant therapy, but recommendations for surgical margins after neoadjuvant therapy remain ambiguous and lack prospective evidence. In breast cancer with neoadjuvant therapy, breast conservation is realized among those who are initially breast-conserving surgery-ineligible and downstaged to be breast-conserving surgery-eligible; however, clinical evidence shows that a multifocal pattern of residual disease after neoadjuvant therapy predicts a higher rate of local-regional recurrence^25,32^. The pathological regression pattern of neoadjuvant therapy should be described in this regard. In our trial, the main pathological regression pattern was multifocal regression, in 92.9% (13/14) of patients; meanwhile, unit residual foci were found only in one non-MPR patient. All patients in the MPR group (except those with pCR) showed a multifocal regression pattern, and in some patients, residual tumor microfoci were found away from the main tumor epicenter, even near the tumor margins. The primary tumor looked melted in situ after NAICT. In this condition, the narrowing scope of tumor resection carries the risk of residual tumor cells after NAICT in OSCC. And more clinical evidence is urgently needed to determine the scope of tumor resection after neoadjuvant therapy in OSCC.

Identifying response biomarkers is important for selecting the appropriate candidates for neoadjuvant therapy and avoiding unnecessary treatment-related AEs in patients who cannot benefit. By comparing pre-NAICT and post-NAICT TIME characteristics, we found that post-NAICT tumor samples showed more TLS than pre-NAICT samples, and the difference was more significant in the MPR group. Previous reports have described the correlation between the presence of TLS and the clinical benefit of immunotherapy^33,34^, and we preliminarily explored whether TLS formation may predict the pathological response to NAICT in LAOSCC. From previous anti-PD-1 trials, there is evidence that PD-L1 expression levels in the primary tumor may indicate clinical benefit. This led to the 2019 approval of pembrolizumab as first-line therapy in patients with HNSCC whose tumors had CPS greater than or equal to 1^13,35^. In our trial, all the four patients with CPS greater than 10 achieved MPR. Although patients with CPS greater than or equal to 1 had a higher MPR rate than those with CPS less than 1, the difference between them was not statistically significant. Other biomarkers validated in solid tumor, including TMB and TIME^36,37^, were also tested in our trial, but we found insufficient evidence.

In conclusion, NAICT with the TTP protocol in LAROSCC is well tolerated with no obstruction on subsequent surgery, and the MPR rate is promising in terms of pathological response. Although the limited sample size precludes exploring predictive biomarkers, the satisfying safety and pathological response results suggest that further randomized trials with NAICT in LAROSCC are well recommended.

## Ethical approval

This study followed the ethical guidelines of the Declaration of Helsinki and was approved by the Institutional Ethics Committee, Ninth People's Hospital, Shanghai Jiao Tong University School of Medicine (SH9H-2020-T93-2). This study was registered at clinicaltrials.gov, (registration number: NCT04473716, Hyperlink：https://clinicaltrials.gov/ct2/show/NCT04473716?term=NCT04473716&draw=2&rank=1.

## Sources of funding

This work was supported by the National Natural Science Foundation of China [Grant Numbers 81972525, 82172734, 82103043], the Science and Technology Commission of Shanghai Municipality [Grant Number 21Y21900300] and Beijing Xisike Clinical Oncology Research Foundation [grant number YYoung-2022-0218].

## Authors contribution

L.Z.: the chief investigator of the trial. L.Z., Q.Z., W.J.: conceptualization, writing - review and editing, supervision; Y.H., J.S., J.L., D.Z., M.D., S.D., Y.T., Q.S., T.Z., Z.Z., X.Z., Y.L., J.L., G.Z.: investigation; Y.H., J.S., J.L., W.S., D.Z., Y.C.: data curation, formal analysis, methodology, roles/writing -original draft; W.J., L.Z.: funding acquisition. All authors read and approved the final manuscript. Yingying Huang, Jingjing Sun, and Jun Li contributed equally to this paper.

## Conflicts of interest disclosure

All authors have no conflicts of interest to declare.

## Research registration unique identifying number (UIN)

Registration number: NCT04473716 (clinicaltrials.gov).

## Guarantor

Dr Laiping Zhong, Department of Oral and Maxillofacial-Head and Neck Oncology, Shanghai Ninth People’s Hospital, College of Stomatology, Shanghai Jiao Tong. University School of Medicine. No. 639 Zhizaoju Road, Shanghai 200011, P. R. China. Tel.:+ 86 212 327 1699 5156; E-mail: zhonglp@hotmail.com

## Data availability statement

Most de-identified clinical data of individual patients are available in the manuscript or additional files. Other data can be obtained upon scientifically sound request from the corresponding author at zhonglp@hotmail.com. DNA data have been deposited in the Genome Sequence Archive for Human (https://ngdc.cncb.ac.cn/gsa-human/) under accession codes for HRA003043 (https://ngdc.cncb.ac.cn/bioproject/browse/PRJCA011820). Due to the policy of our hospital, approval from the Clinical Research Unit needs to be obtained before release of patients’ DNA data. Access to DNA data can be obtained for research purposes from the corresponding author at zhonglp@hotmail.com, who will contact the Clinical Research Unit, Ninth People’s Hospital, Shanghai Jiao Tong University School of Medicine. Once access has been granted, the data will be permanently available for the requester.

## Provenance and peer review

Not commissioned, externally peer-reviewed.

## Supplementary Material
