# Supplementary material for: Neoadjuvant immunochemotherapy for locally advanced resectable oral squamous cell carcinoma: a prospective single-arm trial (Illuminate Trial)
Source: Int J Surg. 2023 Jun 5;109(8):2220–7. doi: 10.1097/JS9.0000000000000489 (PMC10442116; doi:10.1097/JS9.0000000000000489)

# Caption for Supplemental Digital Content

# The clinical trial protocol……………………………………………………3-31

## **Additional Study Methods…………………………………………………32-34**

1. Pathological response patterns to neoadjuvant therapy……………………32
2. Multiplex immunofluorescence of tumor-infiltrating lymphocytes evaluation………………………………………………………………32-33
3. Targeted next-generation sequencing (NGS) and genetic analysis…….33-34

## **Supplementary Tables……………………………………………….……35-41**

1. Table 1. Information of 20 patients for baseline demographic and clinical characteristics………………………………………………………….35-36
2. Table 2. All Therapy-related adverse events………………………….37-38
3. Table 3. Detail information of radiographical and pathological response to NAICT in 20 LAOSCC patients………………………………………………………..39
4. Table 4. Pathological response in metastatic lymph nodes…………...…40
5. Table 5. Clinical to pathological downstaging post-NAICT…………….41

## **Supplementary Figures………………………………………………… 42-48**

1. Figure 1. The model diagram and practice of surgical tumor bed determination…………………………………………………………….42
2. Figure 2. Photo of surgically resected lesion during surgery…………….43
3. Figure 3. Schematic diagram showing pathological response pattern…...44
4. Figure 4. The pathological tumor bed in HE staining of post-NAICT slices……………………………………………………………………..45
5. Figure 5. Pathological response pattern analyses………………………..46
6. Figure 6. Comparation of TILs infiltration in biopsy samples between MPR and non-MPR group……………………………………………………………….47
7. Figure 7. Kaplan-Meier curves for overall survival and disease-free survival……………………………………………………………….….48

## The clinical trial protocol

**Neoadjuvant Toripalimab and Albumin Paclitaxel /Cisplatin on Pathological Efficacy in Patients with Locally Advanced Oral Squamous Cell Carcinoma: A single-arm trail**

**Version number/version date:** Version 1.0/ June 15, 2020

**Trial sponsor:** Ninth People's Hospital, Shanghai Jiao Tong University School of Medicine

**Statistical analyst division:** Ninth People's Hospital, Shanghai Jiao Tong University School of Medicine

**Principal investigator:** Lai-ping Zhong

**Trial start time:** July 30, 2020

**Registration number:** NCT04473716 (clinicaltrials.gov)

**Ethical statement:** SH9H-2020-T93-2 (Institutional Ethics Committee of Ninth People’s Hospital, Shanghai Jiao Tong University School of Medicine)

**Signature page of the main investigator (group leader unit)**

I will conscientiously perform my duties as an investigator in accordance with China’s Good Clinical Practice (GCP) and personally participate in or directly guide this clinical trial. I have received the investigator’s manual of the investigational drug in this clinical trial. I have read and understand the preclinical research status of the investigational drug and the research protocol for this clinical trial. I agree to perform relevant duties in accordance with Chinese laws, the Declaration of Helsinki, China’s GCP and this research protocol. Unless measures must be taken to protect the safety, rights and interests of the subjects, I will only make changes to the protocol after notifying the sponsor and obtaining consent and will implement changes after approval by the Ethics Committee. I will be responsible for making clinical medical decisions, ensuring that subjects receive appropriate treatment in a timely manner when adverse events occur during the study and documenting and reporting these adverse events in accordance with relevant national regulations. I promise to document the data in a true, accurate, complete and timely manner in the study database. I will willingly accept supervision and inspections by personnel dispatched by the sponsor and inspections by the drug supervision and management department to ensure the quality of clinical trials. I promise to keep each subject’s personal information and related matters confidential. Before the start of the study, I will provide the principal investigator’s resume, which will be submitted to the ethics committee and to the drug regulatory department.

**Research Institute**: Ninth People's Hospital, Shanghai Jiao Tong University School of Medicine

**Principal Investigator**: Lai-ping Zhong (print) __________________ (signature)

**Date of Signature:** _________________**Contact Number:** ______________

**Address**: Floor 13, Building 1, No. 639, Zhizaoju Road, Shanghai

**Zip Code:** 200011**I. Summary**

**Title:** Neoadjuvant Toripalimab and Albumin Paclitaxel/Cisplatin on Pathologic Response in Patients with Locally Advanced Oral Squamous Cell Carcinoma: A single-arm trail

**Number**: 20200413

**Sponsor:** Ninth People's Hospital, Shanghai Jiao Tong University School of Medicine

**Responsible Party:** Ninth People's Hospital, Shanghai Jiao Tong University School of Medicine

**Principal Investigator:** Lai-ping Zhong

**Indication:** Oral squamous cell carcinoma

**Trial drugs:** Toripalimab (240mg); TP: (albumin paclitaxel 100mg; cisplatin 10mg, 20mg)

**Objective:** To evaluate the safety and pathological response of inductive Toripalimab and albumin paclitaxel /cisplatin (TP) for patients with locally advanced OSCC.

**Subjects:** Patients with primary locally advanced oral squamous cell carcinoma (III, IVA)

**Number of planned participants:** 20 patients

**Inclusion Criteria:**

1. Age: 18-75years old

2. Gender: male and female

3. Eastern Cooperative Oncology Group (ECOG) performance status (PS) score: 0-1

4. Histopathological diagnosis of oral squamous cell carcinoma (including tongue, gingiva, buccal mucosa, floor of mouth, hard palate, and retromolar tissue)

5. Primary tumor with clinical stage of III/IVA (cT1-2/N1-2/M0 or cT3-4a/N0-2/M0, AJCC 2018)

6. Blood routine: white blood cell (WBC)>3,000/mm^3^, hemoglobin>8g/L, platelet>80,000/mm^3^

7. Liver function: alanine amino transferase/aspartate amino transferase (ALAT/ASAT) <2.5 times the normal upper limit, bilirubin <1.5 times the normal upper limit

8. Renal function: serum creatinine <1.5 times the normal upper limit

9. Sign the informed consent form

**Exclusion Criteria:**

1. Unresolved toxic reactions above grade 2 [(Common Terminology Criteria for Adverse Events (CTCAE 5.0)] caused by previous anticancer treatments

2. Known grade 3-4 allergic reaction to any drug in treatment

3. Active severe clinical infection (> National Cancer Institute (NCI)-CTCAE version 5.0 grade 2 infection)

4. Uncontrollable hypertension or active cardiovascular disease, such as cerebrovascular accident (≤6 months), myocardial infraction (≤6 months), unstable angina pectoris, congestive heart failure classified II or above by the New York College of Cardiology (NYHA Appendix 5) or sever arrhythmias that cannot be controlled by drugs or have potential effects on trail

5. Patients with chronic diseases require immunotherapy or hormone therapy

6. Pregnant or lactating women

7. Participation in other clinical trials within 30 days before enrollment

8. Other situations that the investigator considers unsuitable to participate in the trial

**Trial Design:** single-arm, single-center, open label clinical trail

**Trial Procedures:** screening period, treatment and observation period, and follow-up period.

**Screening Period:**

- Medical history: including medical history, personal history and medication history;
- Physical examination: including height, weight, blood pressure and heart rate
- Tumor assessment: contrast-enhanced CT, MRI scan and other means can be used. The results of tumor evaluation 21 days prior to screening can be used for baseline screening
- ECOG PS score
- Quality of life questionnaire
- Blood routine
- Blood biochemical
- Blood electrolyte: potassium, sodium, chlorine
- Stool routine and occult blood test
- Urine routine
- [Coagulation](javascript:;) [function](javascript:;)
- Standard 12-lead electrocardiogram
- Pregnancy test: suitable for women of childbearing age
- Virus markers: Hepatitis B, Hepatitis C, HIV TRUST

Eligible patients were enrolled and began receiving drugs treatment.

**Treatment and Observation Period:**

**Neoadjuvant Therapy**

Dose and administration method: The participants received two cycles of intravenous Toripalimab (anti-PD1 inhibitor, 240 mg) every three-week, albumin paclitaxel (260mg/ m2), cisplatin (75mg/ m2) every three week. If the disease progresses or unacceptable toxic reaction occurs during the period, the neoadjuvant therapy is terminated in advance and receive surgery.

To evaluate the safety of immunological and chemotherapeutic drugs, and to evaluate the pathologic efficacy by the changes of tumor cells percentage in pre-treatment biopsy and pathological specimens after surgery.

During this period, at the beginning of each treatment cycle, the participants will be required to visit the hospital to report concomitant medications, evaluate adverse events and compliance, and finish the following laboratory examinations:

- Blood routine
- ECOG PS score
- Urine routine
- Blood biochemical
- Blood electrolyte: potassium, sodium, chlorine
- [Coagulation](javascript:;) [function](javascript:;)
- Other indicators that investigator consider to be examined
- Physical examination: including height, weight, blood pressure and heart rate

Frequency of examination: the above evaluation must be completed within ±5 days of the first day of each treatment cycle, and the investigators must review the results of laboratory tests before administering the drug. Contrast-enhanced CT or MRI of oral and maxillofacial neck was evaluated within 2 weeks after the end of the second cycle of neoadjuvant therapy. If disease progresses during neoadjuvant therapy, the patients will receive perioperative prepared. If serious adverse events occur during neoadjuvant therapy, the treatment should be discontinued according to the judgment of the investigator.

**Surgical Treatment Stage (within 2 weeks after the complete of neoadjuvant therapy)**

The participants will receive surgery within two weeks after the complete of neoadjuvant therapy. The perioperative examination including physical examination, vital signs, blood routine, urine routine, blood biochemistry, blood electrolytes, surgical records, assessment of pathological efficacy, adverse reactions during surgical treatment, and concomitant medication for the treatment of adverse reactions.

**Postoperative Radiotherapy/Chemoradiotherapy (within 6 weeks after surgery)**

Complete blood routine, urine routine, blood biochemistry, blood electrolytes, radiotherapy regimen records, chemotherapy drugs records, concomitant medication records and adverse events during this period. If necessary, tumor imaging evaluations can be performed.

**Follow-up Period:**

The follow-up period is two years. Contrast-enhanced CT and/or MRI will be performed at least every six months to evaluate the tumor and record the survival status and local tumor recurrence of patients. The first follow-up will be within 30 days after the completion of treatment, and then follow-up will be conducted every three months to record tumor information, survival information, ECOG score, concomitant medication/treatment, and adverse events. Enhanced CT and/or MRI for oral and maxillofacial and neck regions and chest CT will be performed every six months after the treatment for imaging evaluation; Additionally, if the physicians find the risk of tumor recurrence or metastasis is revealed during follow-up, CT and/or MRI will be performed.

**Evaluation index**

**Primary endpoints:**

- **Pathologic Efficacy:** The percentage of residual tumor cells in tumor bed of pathological specimens after surgery.
- **Adverse Events:** To evaluate the adverse events during the study period according to the NCI-CTCAE (National Cancer Institute Common Terminology Criteria for Adverse Events), version 5.0.

**Secondary Endpoints:** two-year overall survival rate, local tumor recurrence rate, graded adverse events

Safety Evaluation: To evaluate the adverse events during the study period according to the NCI-CTCAE, version 5.0; graded adverse events (calculate the incidence of adverse events and serious adverse events)

**II.** **Purpose of the trial**

To conduct a single-arm clinical trial to investigate the pathologic efficacy and safety of neoadjuvant Toripalimab and albumin paclitaxel /cisplatin for patients with locally advanced OSCC. This trial will lay a foundation for the further randomized controlled trials.

**III. Trial Design**

Single-center, single-arm, open-label clinical trial.

**IV. Selection of Participants**

**1. Inclusion criteria: see previous content.**

**2. Exclusion criteria: see previous content.**

**3. Criteria for termination of the trial:**

Termination of the trial refers to the early termination of the treatment specified in the protocol during the clinical trial. The main purpose is to protect the rights and interests of the participants, ensure the quality of the trial and to avoid unnecessary economic losses.

If one of the following conditions is met, the trial will be terminated:

- Serious safety problems during the trial
- There are major errors in the clinical trial protocol making it difficult to evaluate the endpoints, or a well-designed protocol with significant deviations in implementation, making it difficult to evaluate the endpoints if the trial continues
- The investigator requires termination (such as funding reasons, management reasons, etc.)
- The administrative department or the ethics committee require termination
- Investigators consider continuing the trial may harm the interests of the subjects

**4. Withdrawal from the trial:**

Participants have the right to withdraw from the trial at any time for any reason. Investigators should contact the participants via telephone or follow-up visits or through their relatives to fully understand the reasons for withdrawal and record on the case report form (CRF).

The investigators also have the rights to decide whether withdraw from the trial when recurrence of pre-existing disease, the occurrence of serious adverse events, violation of the treatment protocol, poor compliance of the participants, taking other drugs during the trial that interfere with the efficacy evaluations, and management issues or other reasons. As too many participants withdraw will lead to the trial results unreliable, so unnecessary withdraw should be avoided. If a patient withdraws from the study due to the adverse events or abnormal laboratory test results, it should be recorded in the CRF. Participants who withdraw early cannot be replaced by other participants.

**V. Experimental drugs and management**

**1.** **Trial drug 1: Toripalimab**

Manufacturer: Suzhou Zhonghe Bio-pharmaceutical Technology Co., LTD

Dosage form: lyophilized powder

Specification: 240mg

Usage: intravenous injection

Expiration date: three years

Storage conditions: sealed, protected from light and placed in 2-8 °C medical refrigerator

1. **Trial drug 2: Albumin paclitaxel**

Manufacturer: Qilu Pharmaceutical(Hainan) Co., Ltd

Dosage form: lyophilized powder

Specification: 100 mg

Usage: intravenous injection

Expiration date: two years

Storage conditions: sealed, protected from light, and stored 20~30 °C

1. **Trial drug 3: Cisplatin**

Manufacturer: Qilu Pharmaceutical Co., Ltd

Dosage form: lyophilized powder

Specification: 10 mg or 20 mg

Usage: intravenous injection

Expiration date: two years

Storage conditions: protected from light, airtight storage

**4. Administration method**

Toripalimab (anti-PD1 inhibitor, 240 mg) every three week; TP every three week: albumin paclitaxel (260mg/ m^2), cisplatin (75mg/ m^2) at an interval of one hour.

There will be two cycles of neoadjuvant therapy, and then the participants receive radical surgery and postoperative radiotherapy/ chemoradiotherapy. The participants use the drugs until the treatment termination criteria specified in the study appear.

**5. Concomitant Medication**

To collect the medication history of the subjects within the 14 days prior to screening and the concomitant treatment information during the entire trial period. And record all concomitant medications and treatment methods in the CRF in detail, and note the reason, dose, and time of medication.

- The participants will not be allowed to use any antitumor drugs other than the trial drugs during the entire trial
- Researchers can take appropriate supportive treatment after evaluating the relationship between adverse events and medication. The start and duration of supportive treatment, e.g., antiemetics, antidiarrheals, antipyretics, antihistamines, analgesics, antibiotics, and other blood products, will be documented in the CRF.
- Researchers should provide maintenance therapy for underlying conditions (such as hypertension, diabetes, etc.). And fully record medications and reasons for administration in the concomitant medication section of CRF.
- During the trial period, participants will be prohibited from using CFDA-approved modern Chinese medicine preparations and immunomodulatory agents for the treatment of oral cancer (including but not limited to interferon, interleukin-2, thymosin, etc.)

During the trial period, participants will not be allowed to receive any local treatment for oral cancer lesions, and other systemic antitumor therapies, such as chemotherapy, molecular targeted therapy, steroid therapy, immunotherapy, and traditional Chinese medicine treatment will not be allowed.

**6. Drug distribution and management**

All drugs in the trial will be managed by specialized drug administrator to ensure that the drugs are only used for qualified subjects of the clinical trial. It is prohibited to distribute the trial drugs to non-enrolled individuals. The clinical research associate (CRA) is responsible for monitoring the supply, use, storage and the disposal process of residual drugs in the trial.

The trial drugs will be stored in a locked medicine cabinet as required.

The investigators are responsible for the inventory, verification and recording of the trial drugs. The investigators or designated personnel must keep a record of the number of drugs, including the missing drugs during the entire trial process, i.e., the trial drugs will be kept and distributed by a designated person in the clinical trial institute, and the process of accepting, receiving and returning will be recorded in detail.

Once the study is completed, if all unused drugs that are not destroyed locally by authorized by the sponsor, the CRA will retrieve the drugs along with the corresponding use records.

**VI.** **Study Procedure**

In this study, neoadjuvant therapy use Toripalimab combine with TP chemotherapy, participants who meet the protocol will be treated with the trial drugs. The entire trial will be divided into screening period, treatment and observation period, and follow-up period.

**Screening Period:**

- Medical history: including medical history, personal history and medication history;
- Physical examination: including height, weight, blood pressure and heart rate
- Tumor assessment: CT, MRI scan and other means can be used. The results of tumor evaluation 21 days prior to screening can be used for baseline screening
- ECOG PS score
- Quality of life questionnaire
- Blood routine
- Blood biochemical
- Blood electrolyte: potassium, sodium, chlorine
- Stool routine and occult blood test
- Urine routine
- [Coagulation](javascript:;) [function](javascript:;)
- Standard 12-lead electrocardiogram
- Pregnancy test: suitable for women of childbearing age
- Virus markers: Hepatitis B, Hepatitis C, HIV TRUST

To select eligible patients to the enrollment process and began receiving drug treatment.

**Treatment and Observation Period:**

**Neoadjuvant therapy**

Dose and administration method: The participants received two cycles of intravenous Toripalimab (anti-PD1 inhibitor, 240 mg) every three week, albumin paclitaxel (260mg/m^2^), cisplatin (75mg/m^2^) every three week. If the disease progresses or unacceptable toxic reaction occurs during the period, the neoadjuvant therapy is terminated in advance and receive surgery. After the neoadjuvant therapy, the participants will receive radical surgery and postoperative radiotherapy/ chemoradiotherapy.

Prophylactic medication and protective measures during preoperative neoadjuvant therapy:

1. Antiemetic drugs can be given prophylactically
2. Short-acting or long-acting granulocyte colony stimulating factor (G-CSF), thrombopoietin (TPO), erythropoietin (EPO), and other drugs or component blood transfusion support treatment can be given when hematopoietic suppression more than 2 degrees occurs. These drugs can be used prophylactically in patient who have experienced hematopoietic suppression of more than 3 degrees on previous chemotherapy and have not reduced. The specific doses are as follows:
3. Short-acting G-CSF 5ug/kg, [hypodermic](javascript:;) [injection](javascript:;), once a day; long-acting G-CSF 6mg [hypodermic](javascript:;) [injection](javascript:;), starting from the 3rd-4th day of chemotherapy until the white blood cell count is more than 4.0×10^9^/L
4. TPO 300ug/kg, [hypodermic](javascript:;) [injection](javascript:;), once a day until the platelet count is more than 100×10^9^/L or increases 50×10^9^/L
5. EPO 100IU/kg, [hypodermic](javascript:;) [injection](javascript:;), once a day until hemoglobin is more than 0g/L
6. Hepatoprotective drugs cannot be given prophylactically during the first treatment
7. Anti-hepatitis B virus drugs
8. If thyroid and pancreatic dysfunction occurs in the treatment, antithyroid drugs or hormone or insulin supplementation are required

**Chemotherapy dose adjustment and withdrawal:** In principle, standard dose chemotherapy drugs should be given in the study.

If grade 3 or above (NCI-CTCAE 5.0) non-hematological toxicity (except nausea and vomiting) or grade 4 hematological toxicity (except for grade 4 granulocytopenia without infection and fever) occurs, and subsequent chemotherapy is delayed for more than 1 week despite supportive treatment the next course of chemotherapy drugs should be 75% of the original dose. If the same toxicity occurs again, or the subsequent treatment is delayed for more than 2 weeks due to toxic and side effects, the clinical trial will be withdrawn. For patients older than 65, the researchers can give 75% of the standard dose initially, based on the assessment of physical fitness and comorbidities, and subsequent chemotherapy intensity may be adjusted as described above.

**Adverse reactions management and withdrawal of PD-1antibody:** In principle, standard dose PD-1antibody drugs should be given in the study.

When mild or moderate infusion reaction occurs, slow down or suspend infusion, and monitor patients' vital signs, pulse oxygen and electrocardiogram at the same time, and can give antihistamines (finagan, etc.) for symptomatic treatment. If infusion reaction is not alleviated or severe infusion reaction occurs, the clinical study will be withdrawn. If 1-2 degree of PD-1 antibody related immunotoxicity occurs, to handle refer to the irAE (immune-related adverse events) guidelines (symptomatic treatment such as corticosteroids and immunosuppressants). If there is no remission or more than 3degree toxicity occurs after treatment, the study will be withdrawn.

**Conditions for continued treatment:**

1. If the disease is well controlled, myelosuppression is in recovery stage, neutrophils ≥1.5×10^9^/L, platelet count ≥75×10^9^/L, hemoglobin ≥90g/L, the next course of treatment will be carried out as planned, otherwise the treatment will be delayed for 1 week
2. If the disease is not well controlled and myelosuppression is in recovery stage, it is not recommended to postpone the treatment, and chemotherapy can be scheduled under supportive treatment
3. If treatment is delayed for more than 2 weeks and the criteria are not met, the patient will withdraw from the treatment and record an adverse event. And follow-up will continue according to protocol requires

To evaluate the safety of immunological and chemotherapy drugs, and according to the percentage change of tumor cells in biopsies before treatment and pathological specimens after surgery to evaluate pathological efficacy.

During the period, at the beginning of each treatment cycle, the participants will be required to visit the hospital to report concomitant medications, evaluate adverse events and compliance, and finish the following laboratory examinations:

- Blood routine
- ECOG PS score
- Urine routine
- Blood biochemical
- Blood electrolyte: potassium, sodium, chlorine
- [Coagulation](javascript:;) [function](javascript:;)
- Other indicators that investigator consider to be examined
- Physical examination: including height, weight, blood pressure and heart rate

Frequency of examination: the above evaluation must be completed within ±5 days of the first day of each treatment cycle, and the investigator must review the results of laboratory tests before administering the drug. Enhanced CT or MRI of oral and maxillofacial neck was evaluated within 2 weeks after the end of the second cycle of induction therapy. If disease progresses during neoadjuvant therapy, the patients will receive surgery. If serious adverse events occur during neoadjuvant therapy, the treatment should be discontinued according to the judgment of the investigator.

**Surgical Treatment Stage (within 2 weeks after the complete of neoadjuvant therapy)**

The participants will receive surgery within two weeks after the completed of neoadjuvant therapy. The perioperative examination includes physical examination, vital signs, blood routine, urine routine, blood biochemistry, blood electrolytes, surgical records, assessment of pathological efficacy, adverse reactions during surgical treatment, and concomitant medication for the treatment of adverse reactions.

**Postoperative Radiotherapy/Chemoradiotherapy (within 6 weeks after surgery)**

Complete blood routine, urine routine, blood biochemistry, blood electrolytes, radiotherapy regimen records, chemotherapy drugs records, concomitant medication records and adverse events during this period. If necessary, tumor imaging evaluations can be performed.

**Follow-up Period:**

The follow-up period is two years. CT and/or MRI will be performed at least every six months to evaluate the tumor and record the survival status and local tumor recurrence in the patients. The first follow-up will be within 30 days after the completion of treatment, and then follow-up will be conducted every three months to record tumor information, survival information, ECOG score, concomitant medication/treatment, and adverse events. Enhanced CT and/or MRI for oral and maxillofacial and neck regions and chest CT will be performed every six months after the treatment for imaging evaluation; Additionally, if the physicians find the risk of tumor recurrence or metastasis is revealed during follow-up, CT and/or MRI will be performed.

**Study Flow Chart**

|  | Screening period | Inductive therapy | Surgical treatment | Postoperative treatment | Follow-up period |
| --- | --- | --- | --- | --- | --- |
| examination item | -3w~0w | 0w-6w |  |  | If necessary |
| informed consent | X | - |  |  |  |
| medical history | X | - |  |  |  |
| pegnancy test ^1^ | X | - |  |  |  |
| vital signs | X | once per cycle | X | X |  |
| physical examination | X | once per cycle | X | X |  |
| blood routine | X | once per cycle | X | X |  |
| urine routine | X | once per cycle | X | X |  |
| fecal routine | X | once per cycle | X | X |  |
| blood biochemistry | X | once per cycle | X | X |  |
| coagulation function | X | once per cycle | X | X |  |
| HBV, HCV, HIV, and TRUST | X | - |  |  |  |
| Tumor evaluation ^2^ | X | before surgery | X | X |  |
| 12-lead ECG | X | if necessary | X | if necessary |  |
| cardiac B-ultrasound ^3^ | X | if necessary | X | if necessary |  |
| enhanced CT scan or MRI examination of oral and maxillofacial and neck regions | X | before surgery | X | X |  |
| assign subjects identification number | X | - |  |  |  |
| administration |  | Toripalimab (anti-PD1 inhibitor, 240 mg) and albumin paclitaxel (260mg/m^2^) and cisplatin (75mg/m^2^) every three week  radical surgery  postoperative radiotherapy/ chemoradiotherapy |  |  |  |
| concomitant medication | X | once per cycle | X | X |  |
| record adverse events | X | once per cycle | X | X |  |

Note: 1. Pregnancy test: only applicable to women of childbearing age. 2. Tumor evaluation will be performed using CT and/or MRI, but the same subject must receive the same examination method. 3. B cardiac B-ultrasound to evaluate pericardial effusion.

**VII.** **Trial Endpoint**

**Primary Endpoints:**

- **Pathologic Efficacy:** The percentage of residual tumor cells in tumor bed of pathological specimens after surgery.
- **Adverse Events:** To evaluate the adverse events during the study period according to the NCI-CTCAE, version 5.0.

**Secondary endpoints:** two-year overall survival rate, local tumor recurrence rate

**Safety endpoints:** To evaluate the adverse events during the study period according to the NCI-CTCAE, version 5.0. Graded adverse events, including vital signs, physical examination, laboratory tests, changes in ECOG score, etc., as well as adverse events and serious adverse events, are calculated to the incidence of adverse events and serious adverse events. To use quality of life questionnaire EORTCQLQC30 to evaluate the improvement in quality of life before and after treatment.

**VIII.** **Adverse events and serious adverse events**

**1. Adverse Events**

Adverse events (AEs) refer to any adverse signs, symptoms or medical conditions that occur or aggravate after the use of trial drugs, even if the event is considered unrelated to the trial drugs. The research drugs include experimental drugs and placebos. Medical conditions/diseases that exist before the start of the trial will be recorded as an AE only if aggravate after the use of trial drugs. Abnormal laboratory results will be considered AEs when they cause clinical symptoms or signs, or considered to have significant clinical significance, or require intervention.

In this trial, AEs will be recorded from signing the informed consent form to 30days after the last administration. Any serious AEs that occur 30 days after the termination of the treatment will be reported only when the investigator suspects that such AEs are related to the intervention of this trial.

While the participants stay in the research center, they will be asked regularly about the occurrence of AEs (using neutral questions, such as “how do you feel?”).

The AEs spontaneously reported by the participants during the trial period and/or revealed by responses to questions and the AEs found in the physical examinations, laboratory tests or other evaluations will be recorded in eCRF adverse event form, and note severity (mild, moderate, and severe), onset time, end time, duration, treatment measures (including measures for trial drugs), outcome, relationship with trial drugs, and whether it is a serious adverse event (SAE), etc. will be specified. Adverse events that occur after the signing of informed consent but before the start of treatment should be recorded in the past /medical history part of the eCRF. All adverse events should be followed until they are properly resolved or the condition is stable.

Adverse drug reactions (ADRs) refer to AEs associated with any dose of the trial drugs. All adverse events that the investigators judge to have a reasonable causal relationship with the trial drugs will be considered ADRs.

1.1 Severity of AEs

The severity of AEs will be evaluated by using the CTCAE, version 5.0:

**Grade 1**: mild; asymptomatic or mild, only clinically or diagnostically observable events; no treatment is needed

**Grade 2**: moderate; requires minor, local or non-invasive treatment; age-appropriate limitation in instrumental activities of daily living

**Grade 3**: severe or medically significant but not immediately life-threatening; leading or prolonging hospitalization; disability; limitation of personal activities of daily living

**Grade 4**: life-threatening; require emergency treatment

**Grade 5**: AE-related death

Note: instrumental activities of daily living refer to cooking, buying clothes, using phone, managing finances, etc. Personal activities of daily living include bathing, dressing and undressing, eating, washing, taking medication, etc., not being bedridden.

1.2 Causality assessment

The following terms will be used to describe the causal relationship between AEs and trial drugs:

**Definitely related**: a clinical event includes laboratory abnormalities, the occurrence of which has a reasonable sequential relationship with the application of trial drugs and cannot be explained by concomitant diseases or other drugs. The drug withdrawal reaction is clinically reasonable. This event must have a positive correlation with the trial drugs in terms of pharmacological or clinical manifestations and reappear when the drug is administered again.

**Most likely related**: a clinical event includes laboratory abnormalities, the occurrence of which has a reasonable sequential relationship with the application of trial drugs and is unlikely to be caused by concomitant diseases or other drugs. The drug withdrawal reaction is clinically reasonable. This event does not necessarily occur when the drug is administered again.

**May be related**: a certain clinical event includes laboratory abnormalities, the occurrence of which has a reasonable sequential relationship with the application of trial drugs but also can be explained by concomitant diseases or other drugs. Drug withdrawal information can be missing or unclear.

**May be unrelated**: a certain clinical event includes laboratory abnormalities, the occurrence of which has a sequential relationship with the application of trial drugs but may not have a causal relationship with the trial drugs. Other drugs or diseases can provide a reasonable explanation for the causal relationship.

**Irrelevant**: AEs that do not meet any of the above criteria.

1.3 The outcome of AEs

The following terms are used to describe the outcome of AEs:

- Recovery
- Recovering
- Recovery with sequelae
- Not recovered
- Death
- Unknown

1.4 Follow-up of AEs

If an AE occurs at the end of the study evaluation or continues, the investigator will continue to follow-up until the event disappears or the condition is stable.

**2. Sever Adverse Events (SAEs)**

SAEs are defined as events that lead to at least one of the following outcomes:

- Death
- Life-threatening
- Need to be hospitalized or prolong hospitalization
- Persistent or significant disability/inability to work
- Congenital anomalies/birth defects

Other important medical events: although not immediately life-threatening or a direct cause of death or hospitalization, they may endanger the health of subjects or may require medical intervention to prevent the occurrence of any important AE/ADR.

Researchers should immediately take appropriate treatment measures for participants with SAEs to ensure their safety and record the treatment measures and the progress of the events. If the SAE is determined by the investigator to be related to the trial drugs, the sponsor will provide certain financial compensation in accordance with the relevant national laws and regulations.

For any serious adverse events that occur after the subject signs the informed consent form until the termination of the trial within 30 days, regardless of whether it is related to the trial drugs or not, the investigators must report it to the sonsor unit within 24 hours, and report to the Ethics Committee and the National Medical Products Administration (NMPA) by fax as required by GCP. SAE that occurs 30 days after the termination of the trial will be reported only when the investigators consider that such SAE is related to the trial drugs.

Investigators should collect all the information of SAEs and record them in SAE report form. Investigators should evaluate the correlation between SAEs and trial drugs, complete and sign the SAE report form, and fax the completed and signed report form to the sponsor within 24 hours. The original SAE report form and fax confirmation form should be in the clinical trial center.

Within 24 hours after receiving follow-up information, investigator must report recurrence, complications, or progression of the original SAE as a follow-up event of the original event. SAEs that occur at different time are considered completely unrelated to previously reported SAEs should be reported separately as new SAEs. The follow-up report should explain whether the AEs are resolved or continue, whether the treatment is needed and how, whether unblinding is necessary, and whether the subject continue the trial. The sponsor unit can raise questions to clarify the details of SAEs.

**IX.** **Statistical Analysis**

To use SPSS20.0 software to analyze the efficacy and safety.

- - - 1. **Efficacy analysis**

Primary endpoints

- Safety
- Pathological efficacy

Secondary endpoints

- 2-year survival rate
- Local recurrence rate of tumor
  - - 1. **Safety analysis**
- Incidence of adverse events
- The subsystem lists the frequency of adverse events and calculates the percentage
- A list of all adverse events
- A list of all cases of adverse reactions
- Evaluate the adverse events during the treatment and observation period in accordance with NCI-CTCAE 5.0
  - - 1. Complete quality of life questionnaire EORTCQLQC30 to evaluate the scores of subjects in various areas during treatment and evaluate the improvement of quality of life.

**X. Clinical trial Management**

**1.** **Approval of the ethics committee**

The design, implementation and report of this clinical trial is in accordance with the management regulations of GCP, the current Helsinki Declaration, the relevant regulations of the NMPA and the tips of the ethics committee.

The investigator should obtain written approval of the ethics committee for the trial protocol, informed consent, subject recruitment procedures and other written information that will be provided to the subjects before the start of the trial. During the trial period, if there are any new amendments of the trial protocol and informed consent form, should obtain written approval from the ethics review committee again before implementation.

The composition and operation of the ethics committee should comply with national regulations.

1. **Informed consent**

The investigators are responsible for explaining to each subject the clinical trial background, the characteristics of the trial drug, the trial protocol, other treatment measures for related diseases, as well as the benefits and risks of participating in the trial. And obtain written informed consent form signed by each subject or his/her legal representative before each subject participates in the trial (before the screening examination).

The informed consent text will include the following content: the purpose of the trial, the name and the characteristics of the trial drugs, the ADRs, the dosage, the frequency, blood sample collection, the trial procedures, the obligations of the subjects, the risks, inconveniences and compensation for the subjects to participate in the trial; treatment and appropriate compensation to the subjects in the event of trial-related damage; access to the trial data, and confidentiality of subject information, etc.

The informed consent text should be written in a language that the subjects can read. The informed consent form should obtain written approval by the ethics committee. The investigators should sign their signature and date during each subject or his/her legal representative carry out the informed consent. The investigator and the subject will each receive a copy of the informed consent form. If important new information related to the trial drug is found, the informed consent form will be modified in writing and sent to the ethics committee for approval, and then obtain consent again from each subject by the aforementioned procedure as above mentioned.

**3. Data management**

**3.1 Database** **development**

An electronic data acquisition system will be used for data entry and data management in this clinical trial. A database administrator will develop a database based on the protocol and eCRF samples and compile a data entry quality control program according to requirements.

An electronic data capture (EDC) system will be officially launched after testing by the database administrator, data administrator, investigator, clinical research coordinator (CRC), CRA, and medical personnel, etc.

**3.2 Data entry**

Before the start of the trial, the data administrator will provide training for the users of the EDC system, like investigators and the CRC. And the investigators or the CRC will enter the data in real time after startup.

**3.3 Data query**

After the investigators or the CRC complete data entry, the CRA will conduct original data approval online. The CRA, medical staff, and data administrator will verify the data and send data queries online. After sending the queries, the investigators will verify the original data and resolve questions online.

**3.4 Medical coding**

All AEs reported in the clinical trial will be medically coded using the “ICH International Medical terms Dictionary”. Medical coding should be completed before the database is locked.

**3.5 Data management report**

When all the data are cleaned up and the database quality control rate meets requirements, the data administrator will compile data management report based on the protocol and actual operation of the project.

**3.6 Database locking**

The locked data file will not be able to be changed unilaterally. If found problems after database locked, need to be corrected in the statistical analysis feedback query form after confirmation by the principal investigator, statisticians, data administrators and sponsors and written signature for the record.

**4. Trial supervision**

The sponsor will arrange CRA to visit the research institute regularly to conduct clinical supervision. The investigators should actively cooperate with the CRA and allow CRA to have direct access to all the documents related to the trial, such as eCRF, complete original documents and investigator documents. In addition, investigators should answer questions from CRA personnel and correct data in eCRF.

The following specific content will be included in CRA supervision:

- Confirm that the trial center is appropriately equipped before the trial starts, including personnel allocation and training, various examinations related to the trial, a well-equipped laboratory, and good working conditions, that the number of subjects are sufficient, and investigators are familiar with the requirements of the trial protocol.
- To supervise the research institute and investigators before, during and after the trial, confirm that informed consent has been obtained from all subjects before the trial, know the enrollment rate of subjects and the progress status of the trial, and supervise the implementation of the trial is in strict accordance with the trial protocol and GCP.
- Confirm that all data records are correct and complete, all case reports are completed correctly and consistent with the original data. And all errors or omissions have been corrected or noted and the investigators signed and dated.
- Confirm that all AEs are recorded, and SAEs are reported and recorded within the specified time.
- Verify whether the trial drugs are supplied, stored, distributed, and retrieved in accordance with relevant regulations and record accordingly.
- Assist the investigators to make necessary notification and application, report the trial data and results to the sponsor.

**5**. **Audit and inspection**

In addition to regular supervision procedures, the sponsor’s quality assurance department may also check whether the trial is conducted in accordance with GCP and the sponsor’s standard operating procedure (SOP) at any time. During or after the trial, the relevant state departments may also conduct inspections.

**6. Revision of the protocol**

The implementation of this trial should completely follow the trial protocol, GCP, the current Helsinki Declaration, the relevant regulations of CFDA and the tips of the ethics committee. During the implementation of the trial, any changes or additions to the protocol must be in the form of written protocol revision. The revised protocol can only be implemented after approval by the ethics committee.

**7. Termination of the trial**

The sponsor has the right to terminate this trial at any time for any reason. If it is necessary to terminate the trial, the investigators should be informed of the steps to follow to ensure the interests of the subjects are fully protected. The investigators are responsible for reporting the early termination of the trial to the ethics committee.

**8. Confidentiality**

The materials provided by the sponsor to the investigators is nonpublic, must be kept confidential and cannot be released to any organization or institution that is not directly involved in the trial.

Investigators must ensure the anonymity of subjects. The clinical trial should follow the relevant regulations of GCP to protect the subjects’ data. The medical data and lifestyles of all subjects will be processed by computer and will only be transferred to the sponsor or CFDA authorities under the guarantee of high confidentiality.

**XI. Quality control and quality assurance**

The clinical trial institute is recognized by the CFDA with clinical trial conditions of a drug clinical trial institution.

Investigators will be trained in clinical trials, obtain appropriate qualifications and work under the guidance of senior professionals.

To ensure the clinical ward meet the standardized requirements before the trial, to ensure the rescue equipment is fully equipped.

The drugs are given to subjects by professional nursing staffs and know the use of the drugs in detail to ensure compliance of the participants.

Must strictly follow the trial protocol, and truthfully fill in eCRF.

The CRA should follow SOPs, supervise the progress of clinical trials, confirm that all data records and reports are correct and complete, and all eCRF are filled in correctly and consistent with the original data to ensure that the trial processed in accordance with the clinical trial protocol.

Once SAE occurs, the sponsor will temporarily suspend the trial if necessary.

Each institute participating in the trial will be inspected by the sponsor and the drug supervision and administration department. Importantly, investigators and their related personnel should provide time and convenience for inspecting and auditing.

**XII. Research progress**

The enrollment period of this trial is expected last for 8 months, and the study period will be 10 months.

Date of enrollment of the first patient/start of the trial: June 1, 2020

Date of enrollment of the last patient: December 31, 2020

Date of the last patient released from the trial/end of the trial: June 30, 2021

Planned database locking date: June 30, 2023

Estimated report date: September 30, 2023

**XIII.** **Compliance with the trial protocol**

The investigators promise to do their best to avoid protocol violations. If the investigator believes that a certain protocol deviation can improve the implementation of the trial, then protocol revisions will be considered, but revisions can only be implemented after approval by the Medical Ethics Committee. All major protocol violations will be documented and reported in the clinical trial report.

**Appendix---- Clavien-Dindo Grade**

| Grade I | Any deviation from the normal postoperative course without the need for pharmacologic. treatment or surgical, endoscopic, and radiological interventions |
| --- | --- |
|  | Allowed therapeutic regimens are drugs as antiemetics, antipyretics, analgesics, and. diuretics, and electrolytes and physiotherapy. This grade also includes wound infections opened at the bedside |
| Grade II | Requiring pharmacologic treatment with drugs other than such allowed for grade I. complications. Blood transfusions and total parenteral nutrition are also included |
| Grade III  IIIa  IIIb | Requiring surgical, endoscopic, or radiological intervention |
|  | Intervention not under general anesthesia |
|  | Intervention under general anesthesia |
| Grade IV | Life-threatening complication (including CNS complications)* requiring IC/ICU management |
| IVa | Single organ dysfunction (including dialysis) |
| IVb | Multiorgan dysfunction |
| Grade V | Death of a patient |
| *Brain hemorrhage, ischemic stroke, or subarrachnoidal bleeding, but excluding transient ischemic attacks.  CNS, central nervous system; IC, intermediate care; ICU, intensive care unit | |

## Additional Study Methods

*Pathological response patterns to neoadjuvant therapy*

Taking breast cancer as a reference, pathological response patterns to neoadjuvant therapy were classified as unifocal or multifocal regression [24]. As shown in Supplementary Figure 3, unifocal regression is characterized by concentric regression with only one residual tumor lesion, and multifocal regression is characterized by scattered residual lesions that might have a pattern similar to melted ice.

*Multiplex immunofluorescence of tumor-infiltrating lymphocytes evaluation*

The Akoya OPAL Polaris 7-Color Automation IHC kit (NEL871001KT) was used. FFPE tumor slides were deparaffinized in a BOND RX system (Leica Biosystems) and then incubated sequentially with primary antibodies targeting CD163 (Abcam, ab182422, 1:500), CD68 (Abcam, ab213363, 1:1000), PD-1 (CST, D4W2J, 86163S, 1:200), CD3 (Dako, A0452), CD4 (Abcam, ab133616, 1:100), CD8 (Abcam, ab178089, 1:100), CD56 (Abcam, ab75813, 1:100), CD20 (Dako, L26, IR604), FOXP3 (Abcam, ab20034, 1:100) and Pan-CK (Abcam, ab7753, 1:100) (Akoya Biosciences). Then followed by incubating with secondary antibodies and corresponding reactive Opal fluorophores. Nuclei acids were stained with DAPI. Slides incubated with primary and secondary antibodies without fluorophores were used as negative controls.

After staining, slides were scanned using a Vectra Polaris Quantitative Pathology Imaging System (Akoya Biosciences) at 20 nm wavelength intervals from 440 nm to 780 nm with a fixed exposure time and an absolute magnification of ×200. All scans for each slide were then superimposed to obtain a single image. Multilayer images were imported to in Form v.2.4.8 (Akoya Biosciences) for quantitative image analysis. Tumor parenchyma and stroma were differentiated by Pan-CK staining. The quantities of various cell populations were expressed as the number of stained cells per square millimeter in all nucleated cells.

*Targeted next-generation sequencing (NGS) and genetic analysis*

FFPE tissue sections were evaluated for tumor cell content using HE staining. Only samples with a tumor cell content of >20% were eligible for subsequent analyses. DNA extracts (30–200 ng) were sheared into 250 bp fragments using an S220 focused-ultrasonicator (Covaris, Woburn, MA, USA). For targeted capture, indexed libraries were subjected to probe-based hybridization with a customized NGS panel targeting 733 cancer-related genes [23].

The captured libraries were loaded onto a NovaSeq 6000 platform (Illumina, San Diego, CA, USA) for 100-bp paired-end sequencing with a mean sequencing depth of 1000. Tumor mutational burden (TMB) was defined as the number of non-synonymous somatic single nucleotide variants (SNVs) and indels in examined coding regions, with driver mutations excluded. All SNVs and indels in the coding regions of targeted genes, including missense, silent, stop gain, stop loss, in-frame, and frameshift mutations, were considered. The “maftools” package was used to examine the genomic landscape. Copy number variation analysis was performed using an in-house developed pipeline. Fold change thresholds of 1.6 and 0.6 in DNA copy number were set as the cutoffs for amplification and deletion, respectively. Key pathway-related genes were visualized, including the HGF, EGFR/RAS/BRAF, CDK, AKT/mTOR/PI3K, FGFR, p53, epigenetics/chromatin remodeling, DNA damage and repair/telomere stability, and NOTCH pathways. Other clinical trial drug targets and TERT promoter hot spot mutations were also shown in the genomic landscape.

# Supplementary Tables

**Supplementary Table 1.** Information of 20 patients for baseline demographic and clinical characteristics

| Patient No. | Demographic Characteristic | | | | | | | Disease Characteristics ^a^ | | | | |
| --- | --- | --- | --- | --- | --- | --- | --- | --- | --- | --- | --- | --- |
|  | Sex | Age  (years) | ECOG PS | Smoking status | Alcohol use | BMI | Primary site | | cT | cN | Clinical stage | CPS |
| 1 | M | 46 | 1 | Current | Current | 16.90 | Mouth floor | | 3 | 1 | III | 3 |
| 2 | F | 70 | 1 | Never | Never | 19.63 | Tongue | | 3 | 0 | III | 20 |
| 3 | M | 52 | 1 | Current | Current | 27.72 | Mouth floor | | 4a | 0 | IVA | <1 |
| 4 | M | 58 | 0 | Former | Former | 21.51 | Mouth floor | | 3 | 0 | III | 15 |
| 5 | M | 63 | 0 | Former | Never | 23.62 | Gingiva | | 3 | 1 | III | 30 |
| 6 | M | 45 | 0 | Never | Current | 30.67 | Retromolar trigone | | 4a | 1 | IVA | 3 |
| 7 | M | 46 | 1 | Current | Current | 22.41 | Retromolar trigone | | 3 | 0 | III | <1 |
| 8 | M | 19 | 0 | Never | Never | 22.88 | Tongue | | 3 | 0 | III | 5 |
| 9 | M | 75 | 0 | Current | Current | 25.35 | Tongue | | 3 | 0 | III | <1 |
| 10 | M | 32 | 0 | Current | Current | 28.70 | Tongue | | 3 | 0 | III | 15 |
| 11 | M | 49 | 1 | Current | Current | 20.76 | Mouth floor | | 3 | 0 | III | <1 |
| 12 | M | 34 | 0 | Current | Current | 23.18 | Tongue | | 3 | 0 | III | 8 |
| 13 | M | 70 | 1 | Current | Current | 20.76 | Mouth floor | | 3 | 0 | III | <1 |
| 14 | M | 64 | 1 | Former | Current | 24.22 | Gingiva | | 4a | 1 | IVA | <1 |
| 15 | F | 57 | 1 | Never | Never | 24.65 | Tongue | | 3 | 0 | III | <1 |
| 16 | M | 62 | 0 | Former | Current | 22.60 | Mouth floor | | 3 | 1 | III | <1 |
| 17 | M | 50 | 1 | Former | Current | 24.70 | Buccal | | 3 | 0 | III | 0 |
| 18 | M | 56 | 1 | Current | Current | 21.80 | Tongue | | 3 | 0 | III | <1 |
| 19 | F | 66 | 1 | Never | Never | 26.23 | Gingiva | | 4a | 1 | IVA | <1 |
| 20 | F | 31 | 0 | Never | Never | 21.77 | Tongue | | 3 | 1 | III | 0 |

^a^ American Joint Committee on Cancer (AJCC), 8th Edition staging.

Abbreviations: BMI, Body Mass Index; CPS, Combined Positive Score; ECOG PS, Eastern Cooperative Oncology Group performance status; F, Female; M, Male.

**Supplementary Table 2.** All Therapy-related adverse events^a^

| **Period** | **AEs** | **Patient No.** | | | | | | | | | | | | | | | | | | | |
| --- | --- | --- | --- | --- | --- | --- | --- | --- | --- | --- | --- | --- | --- | --- | --- | --- | --- | --- | --- | --- | --- |
|  |  | 1 | 2 | 3 | 4 | 5 | 6 | 7 | 8 | 9 | 10 | 11 | 12 | 13 | 14 | 15 | 16 | 17 | 18 | 19 | 20 |
| **NAICT-related AES** | Baldness | G1 | G1 | G1 | G1 | G1 | G1 | G1 | G1 | G1 | G1 | G1 | G1 | G1 | G1 | G1 | G1 | G1 | G1 | G1 | G1 |
|  | Nausea /vomiting |  | G1 |  | G1 |  |  | G1 | G1 |  | G1 | G1 |  | G1 | G1 | G2 | G2 | G1 | G1 | G3 | G1 |
|  | Fatigue |  | G1 | G1 | G1 |  |  |  | G1 | G1 |  | G1 |  |  | G1 | G1 |  | G1 | G1 | G2 | G1 |
|  | Neutropenia |  | G1 | G4 | G1 | G1 | G1 |  | G1 |  | G1 |  |  |  | G1 |  |  |  |  | G3 |  |
|  | Leukopenia |  | G1 | G3 | G1 |  | G1 | G1 | G1 |  |  |  |  |  | G1 |  |  |  |  | G3 |  |
|  | Skin (rash,  dryness, dermatitis) | G1 |  | G3 |  | G1 |  |  | G1 |  |  | G1 |  |  |  | G1 | G1 |  | G1 |  |  |
|  | Diarrhea |  |  | G2 | G1 |  |  | G1 | G1 | G1 | G1 | G1 |  |  |  |  |  | G1 |  |  |  |
|  | Hyperbilirubinemia |  | G1 | G1 |  |  | G1 |  | G1 |  |  | G1 |  |  | G1 |  |  |  | G1 |  |  |
|  | Uric acid elevation | G1 |  | G1 |  |  |  | G1 |  |  | G1 | G1 |  |  | G1 |  | G1 |  |  |  |  |
|  | Hypokalemia |  | G3 |  | G1 |  |  |  |  |  | G1 |  |  | G1 | G1 |  |  |  |  | G2 |  |
|  | Pain (joint, muscle) |  |  |  |  |  |  |  |  |  |  | G1 | G1 | G1 | G1 |  |  | G1 | G1 |  |  |
|  | Proteinuria |  |  |  |  | G1 |  | G1 | G1 |  |  |  |  |  | G1 |  |  | G1 |  | G1 |  |
|  | AST elevation |  |  | G1 |  | G2 |  |  | G1 |  |  |  |  |  |  |  |  |  |  |  |  |
|  | ALT elevation |  |  | G1 |  | G2 |  |  | G1 |  |  |  |  |  |  |  |  |  |  |  |  |
|  | Hyperthyroidism |  |  |  |  |  |  |  | G1 |  |  |  |  |  |  |  |  |  |  |  |  |
| **Surgical-related AEs** | Subcutaneous exudate |  |  | G1 |  |  |  |  |  |  |  |  |  |  | G1 |  |  |  |  |  |  |
|  | Free flap necrosis |  |  |  |  |  |  |  |  |  |  |  |  |  |  |  |  |  | G3 |  |  |
|  | Flap distal partial necrosis |  |  |  |  |  |  |  |  |  |  |  |  |  |  |  |  |  |  | G1 |  |
| **Adjuvant (Chemo)Radiotherapy-related AEs** | Radiation-induced oral mucositis | G1 | G1 | G1 | G1 | G1 | G1 | G1 | G2 | G1 | G1 | G2 | G2 | G2 | G1 | G1 | G1 |  |  | G1 | G1 |
|  | Radiation-induced dermatitis | G1 | G1 | G1 | G1 | G1 | G1 | G1 | G1 | G1 | G1 | G2 | G2 | G1 | G1 | G1 | G1 |  |  | G1 | G1 |
|  | Fever |  |  |  | G1 |  | G1 |  | G1 | G1 |  | G1 | G1 | G1 |  |  |  |  |  |  |  |
|  | Hemoglobin decreased |  |  | G1 |  |  |  | G1 | G1 | G2 |  | G1 |  | G2 |  | G1 |  |  |  |  |  |
|  | Leukopenia |  |  | G1 |  |  | G1 | G1 | G1 |  |  |  | G1 |  | G1 |  |  |  |  |  |  |
|  | Neutropenia |  |  |  |  |  |  | G1 |  |  |  | G1 | G1 |  |  |  |  |  |  |  |  |

^a^Neoadjuvant and adjuvant therapy-related adverse events (AEs) were recorded throughout the study and graded per the NCI-CTCAE v5.0. Surgery-related complications were graded per the Clavien–Dindo Classification

Abbreviations: NAICT, neoadjuvant immunochemotherapy; G1, Grade 1; G2, Grade 2; G3, Grade3; AST, aspartate aminotransferase; ALT, alanine aminotransferase.

**Supplementary Table 3.** Detail information of radiographical and pathological response to NAICT in 20 LAOSCC patients.

| Patient No. | Radiographical Response^a^ | | | | | Pathological Response | |
| --- | --- | --- | --- | --- | --- | --- | --- |
|  | Primary lesion at baseline  (mm) | Primary lesion after NAICT  (mm) | Δ Primary Lesion (mm) | %Δ Primary Lesion (mm) | RECIST 1.1 | RVT (%) | MPR^b^ |
| 1 | 38 | 10.1 | -27.9 | 73.42 | **PR** | 0 | **+ (pCR)** |
| 2 | 20 | 10.9 | -9.1 | 45.50 | **PR** | 0.5 | **+** |
| 3 | 33.3 | 0 | -33.3 | 100.00 | **CR** | 3.5 | **+** |
| 4 | 25.3 | 14 | -11.3 | 44.66 | **PR** | 9.6 | **+** |
| 5 | 42.8 | 21 | -21.8 | 50.93 | **PR** | 3.8 | **+** |
| 6 | 21.8 | 43.3 | 21.5 | -98.62 | **PD** | 100 | **-** |
| 7 | 27.7 | 0 | -27.7 | 100.00 | **CR** | 0 | **+ (pCR)** |
| 8 | 18.8 | 12.6 | -6.2 | 32.98 | **PR** | 36.4 | **-** |
| 9 | 36.7 | 36.7 | 0 | 0.00 | **SD** | 78.3 | **-** |
| 10 | 27.2 | 11.9 | -13.4 | 56.25 | **PR** | 0 | **+ (pCR)** |
| 11 | 46.6 | 32.1 | -14.5 | 31.12 | **PR** | 11.5 | **-** |
| 12 | 36.9 | 15 | -21.9 | 59.35 | **PR** | 0 | **+ (pCR)** |
| 13 | 21.9 | 14.7 | -7.2 | 32.88 | **PR** | 9.4 | **+** |
| 14 | 24.6 | 23.5 | -1.1 | 4.47 | **SD** | 50.8 | **-** |
| 15 | 21.1 | 19.7 | -1.4 | 6.64 | **SD** | 55.6 | **-** |
| 16 | 22.6 | 21.7 | -0.9 | 3.98 | **SD** | 0 | **+ (pCR)** |
| 17 | 14.7 | 9.5 | -5.2 | 35.37 | **PR** | 0 | **+ (pCR)** |
| 18 | 32.2 | 25.5 | -6.7 | 20.81 | **SD** | 8.4 | **+** |
| 19 | 27.6 | 25.7 | -1.9 | 6.88 | **SD** | 82.9 | **-** |
| 20 | 25.9 | 23.4 | -2.5 | 9.65 | **SD** | 44.7 | **-** |

^a^: According to RECIST v1.1.; ^b^: MPR: no more than 10% RVT. +, MPR; -, non-MPR.

Abbreviations: RECIST, Response Evaluation Criteria in Solid Tumors. PR, Partial Response. SD, Stable Disease. PD, Progressive Disease. RVT, Residual Viable Tumor. MPR, Major Pathological Response. pCR, Pathological Complete Response.**Supplementary Table 4.** Pathological response in metastatic lymph nodes.

| Patient No. | ypN stage | Response in lymph nodes | %RVT | MPR ^a^ in primary tumor |
| --- | --- | --- | --- | --- |
| 1 | 1 | Not significant | 100 | + (pCR) |
| 5 | 1 | Necrosis, Multinucleated giant cells, calcification | 14 | + |
| 6 | 3b | Necrosis, Multinucleated giant cells, Calcification; ENE+ | 100; 47.3 | - (PD) |
| 14 | 2b | Necrosis, Multinucleated giant cells, Calcification | 16; 14.1 | - |
| 16 | 1 | Necrosis, Multinucleated giant cells, Calcification | 6.5; 0 | + (pCR) |
| 20 | 2b | Not significant | 100; 100; 100 | - |

^a^: MPR: no more than 10% RVT. +, MPR; -, non-MPR.

**Supplementary Table 5.** Clinical to pathological downstaging post-NAICT.

| Patient No. | Clinical evaluation | | | | | | Pathological evaluation | | |
| --- | --- | --- | --- | --- | --- | --- | --- | --- | --- |
|  | Pre-NAICT | | | Post-NAICT | | | Post-operation | | |
|  | cT | cN | stage | cT | cN | stage | ypT | ypN | stage |
| 1 | 3 | 1 | III | 2 | 1 | III | 0 | 1 | I |
| 2 | 3 | 0 | III | 2 | 0 | II | 1 | 0 | I |
| 3 | 4a | 0 | IVA | 0 | 0 | I | 0 | 0 | I |
| 4 | 3 | 0 | III | 2 | 0 | II | 2 | 0 | II |
| 5 | 3 | 1 | III | 2 | 1 | III | 1 | 1 | III |
| 6 | 4a | 1 | IVA | 4b | 1 | IVB | 4b | 3b | IVB |
| 7 | 3 | 0 | III | 0 | 0 | I | 0 | 0 | I |
| 8 | 3 | 0 | III | 2 | 0 | II | 3 | 0 | III |
| 9 | 3 | 0 | III | 3 | 0 | III | 3 | 0 | III |
| 10 | 3 | 0 | III | 2 | 0 | II | 0 | 0 | I |
| 11 | 3 | 0 | III | 3 | 0 | III | 2 | 0 | II |
| 12 | 3 | 0 | III | 2 | 0 | II | 0 | 0 | III |
| 13 | 3 | 0 | III | 1 | 0 | I | 1 | 0 | I |
| 14 | 4a | 1 | IVA | 4a | 1 | IVA | 4a | 2b | IVA |
| 15 | 3 | 0 | III | 2 | 0 | II | 3 | 0 | III |
| 16 | 3 | 1 | III | 2 | 1 | III | 0 | 1 | III |
| 17 | 3 | 0 | III | 1 | 0 | I | 0 | 0 | I |
| 18 | 3 | 0 | III | 3 | 0 | III | 3 | 0 | III |
| 19 | 4a | 1 | IVA | 4a | 1 | IVA | 4a | 0 | IVA |
| 20 | 3 | 1 | III | 3 | 1 | III | 3 | 2b | IVA |

# Supplementary Figures

**Supplementary Figure 1.** The model diagram and practice of surgical tumor bed determination (Patient No.10). White arrow, dark makers with tattoo inks which are 0.5 cm away from the palpable lesion margins; Dotted yellow line, surgical tumor bed which is 0.5 cm inside the markers; Dotted purple line, surgical margins which are 0.5-1.0 cm away from the markers.


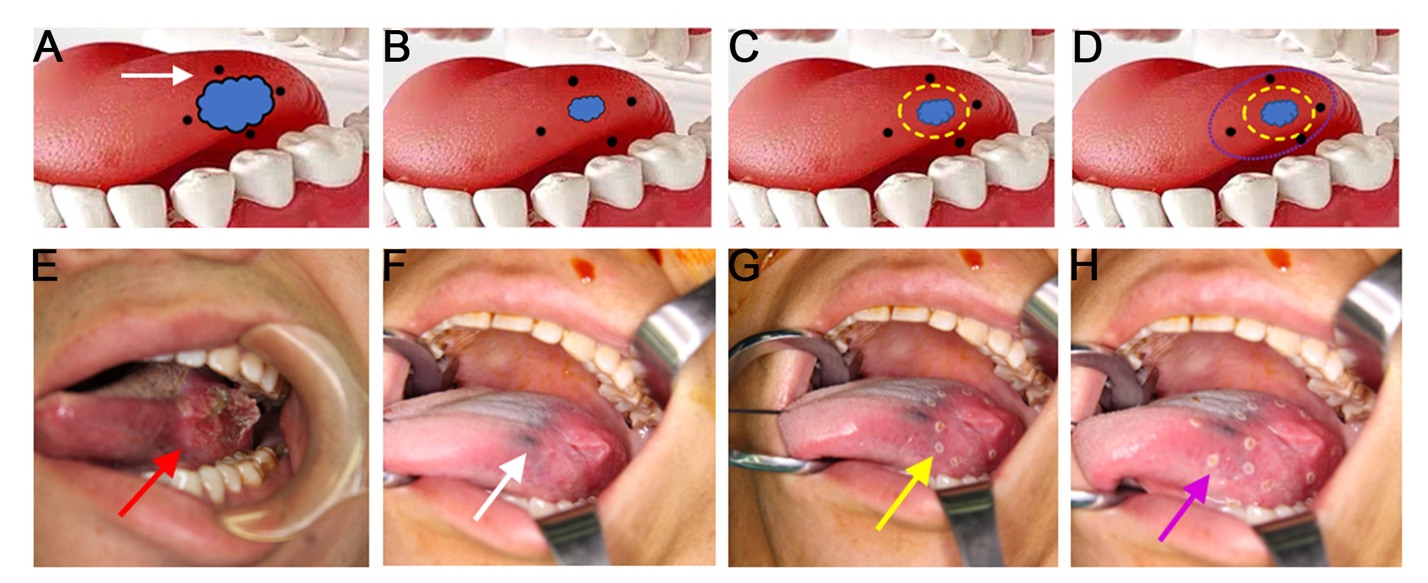


**Supplementary Figure 2.** Photo of surgically resected lesion during surgery (Patient No.10). (A) The resection range of lesion. (B) Surgical tumor bed. (A, Anterior; P, Posterior; S, Superior; I, Inferior)

**
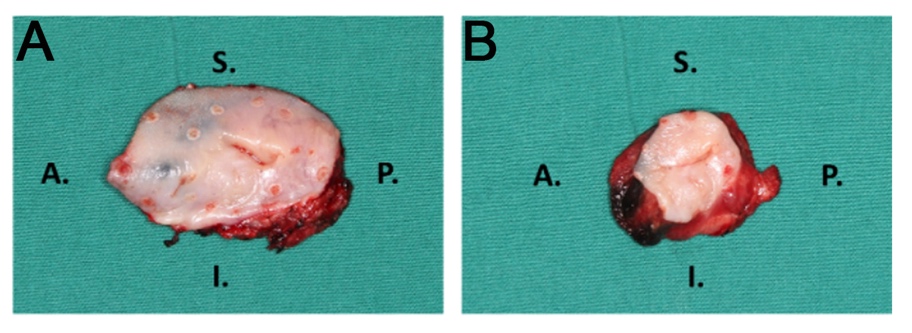
**

**Supplementary Figure 3.** Schematic diagram showing pathological response pattern. (A) Unifocal regression. (B) Multifocal regression. The solid black line, the size of primary lesion at baseline; The dotted black line, the size of primary lesion after neoadjuvant therapy; Gray areas, residual viable tumor.


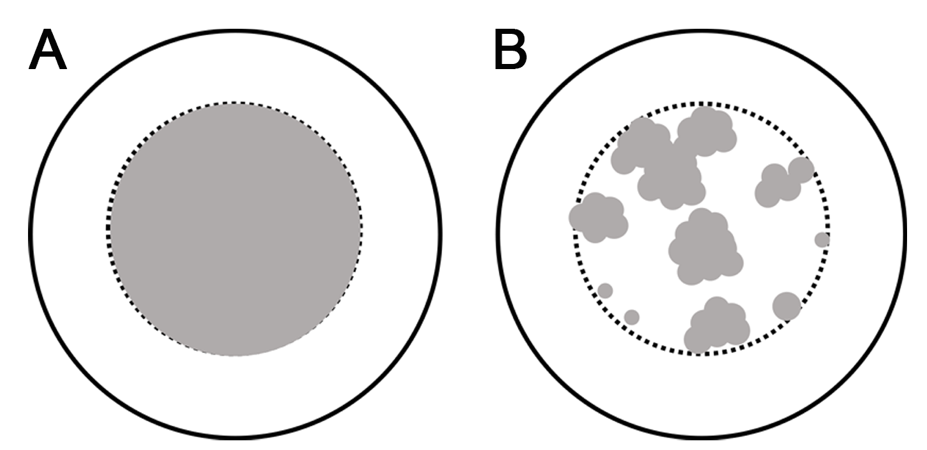


**Supplementary Figure 4.** The pathological tumor bed in HE staining of pos-NAICT slices (Patient No. 2, MPR). The pathological tumor bed was characterized by residual viable tumor, lymphocytes and multinucleated giant cell infiltration, and tissue repair with proliferative fibrosis (the area marked by green line, residual viable tumor; the area marked by yellow line, the pathological tumor bed).


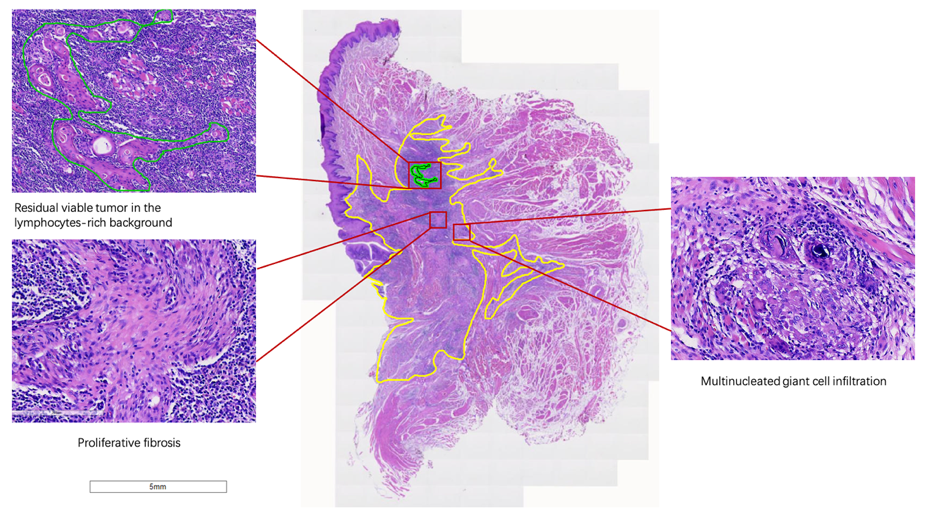


**Supplementary Figure 5.** Pathological response pattern analyses. (A) It showed multifocal regression pattern with multiple residual tumor foci in pathologic tumor bed, and residual tumor microfoci were found away from the main tumor epicenter, even near the tumor margins (<1mm) (Patient No.18, tongue cancer, MPR). (B) Unifocal regression with an isolated residual tumor foci in pathological tumor bed without fibrous tissue separating the foci. The primary lesion shrank little after NAICT with 82.9% RVT (Patient No.19, gingiva cancer, non-MPR) (the area marked by green line, residual viable tumor; the area marked by yellow line, the pathological tumor bed).

**
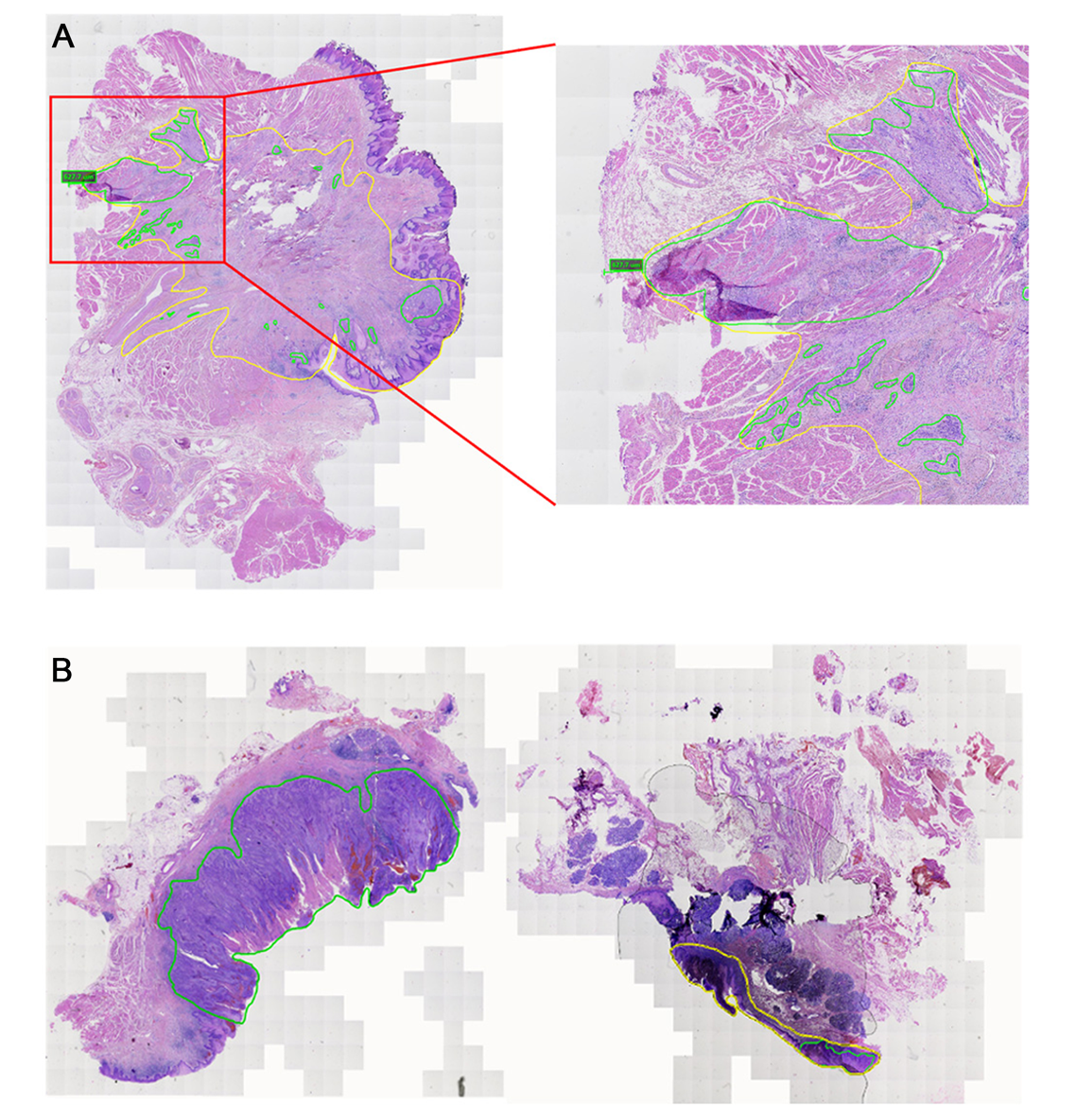
**

**Supplementary Figure 6.** Comparation of TILs infiltration in biopsy samples between MPR and non-MPR group. In pre-NAICT samples, tissues from the MPR group were infiltrated with more CD8+ (P=0.054), CD68+CD163− (P=0.09), cells in the tumour region and CD20+ (P=0.059) and CD3+ (P=0.098) cells in the stroma region.


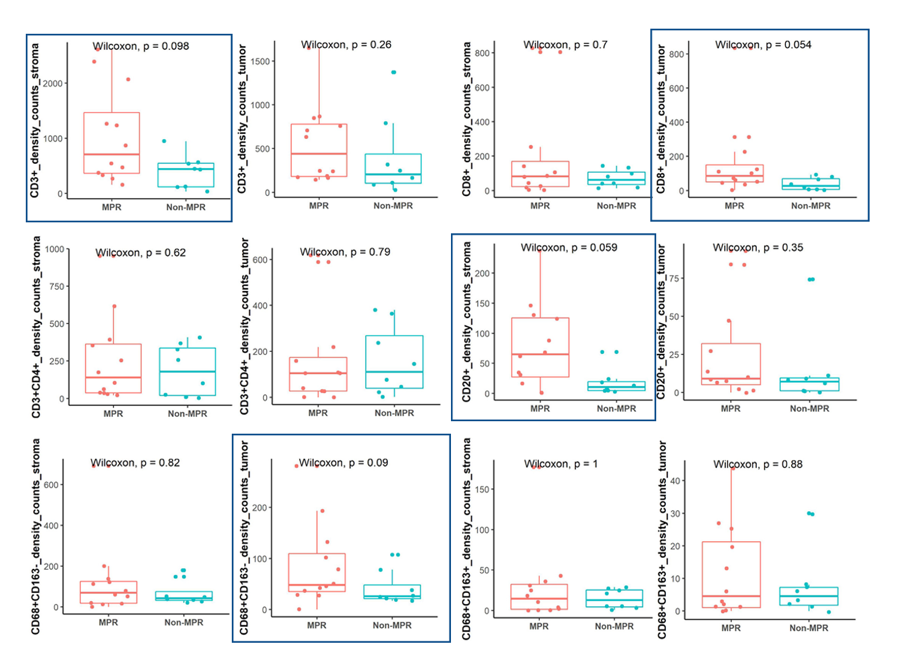


**Supplementary Figure 7.** Kaplan-Meier curves for overall survival (A) and disease-free survival (B).


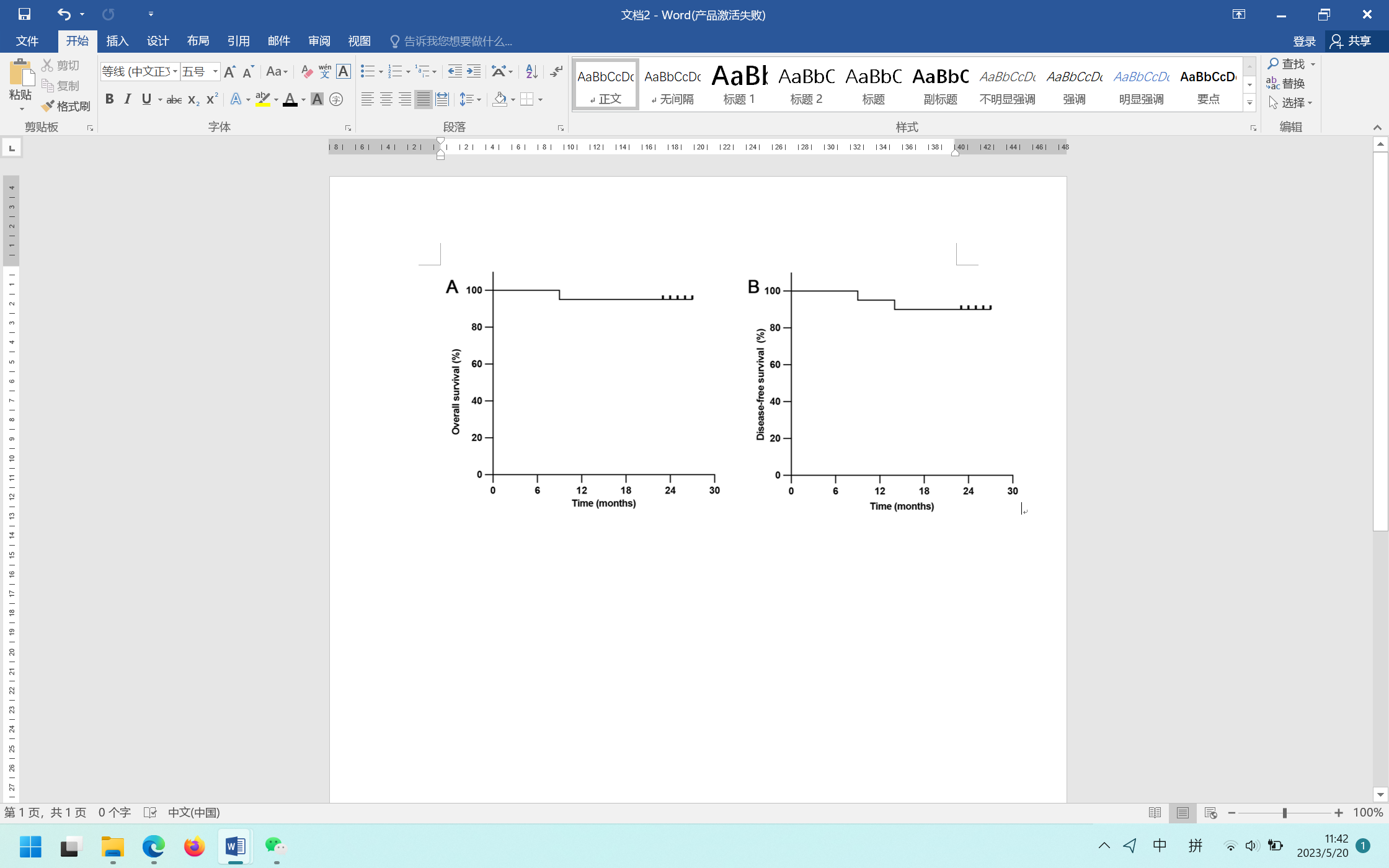

Supplement: Supplementary file 2 [file js9-109-2220-s002.docx]
